# Substituent Effects Control the Biological Activity of Mn(II) Imidazo[1,2-a]pyridine Complexes

**DOI:** 10.3390/molecules31061007

**Published:** 2026-03-17

**Authors:** Magdalena Rydz, Tomasz Mazur, Anna Świtlicka, Urszula K. Komarnicka, Daria Wojtala, Monika K. Lesiów, Agnieszka Kyzioł, Paweł Kędzierski, Dariusz C. Bieńko

**Affiliations:** 1 Faculty of Chemistry, Wroclaw University of Science and Technology, Wybrzeze Wyspianskiego 27, 50-370 Wroclaw, Poland; 2 Department of Crystallography, Institute of Chemistry, University of Silesia, Szkolna 9, 40-006 Katowice, Poland; 3 Faculty of Chemistry, University of Wroclaw, F. Joliot-Curie 14, 50-383 Wroclaw, Poland; 4 Faculty of Chemistry, Jagiellonian University, Gronostajowa 2, 30-387 Krakow, Poland

**Keywords:** manganese(II) complexes, imidazo[1,2-a]pyridine derivatives, thermogravimetric analysis, biological properties, spectroscopic research, molecular docking

## Abstract

Three new Mn(II) complexes with imidazo[1,2-a]pyridine derivatives were synthesized and structurally characterized in a solid state by single crystal X-ray diffraction, FT-IR and Raman spectroscopy, and thermal analyses. The investigated compounds include [Mn(3-Climpy)_2_Cl_2_(MeOH)_2_] (**1**), [Mn(3-Brimpy)_2_Cl_2_(MeOH)_2_] (**2**), and a rare double chloro-bridged coordination polymer [Mn(impy)_2_Cl_2_]_n_ (**3**). Spectroscopic studies were used to assess their potential stability in DMEM (Dulbecco’s Modified Eagle Medium), and encapsulation in Pluronic P-123 micelles improved their solubility in aqueous solution, as well as cellular uptake and selectivity. Biological evaluation revealed negligible cytotoxicity against most cancer and control cell lines, but unexpectedly high activity against pancreatic adenocarcinoma (PANC-1), exceeding that of cisplatin. Complex **2**, bearing a bromine substituent in the imidazole ring, showed the strongest effects, correlating with enhanced intracellular accumulation, reactive oxygen species (ROS) generation, and mitochondrial membrane potential disruption. Molecular docking and protein binding assays demonstrated moderate affinity toward human serum albumin (HSA) and transferrin, whereas DNA interaction was weak and non-damaging. These results highlight the structure–activity relationship of Mn(II) imidazo[1,2-a]pyridine complexes and support their potential as targeted redox-active agents against pancreatic cancer, with polymeric encapsulation providing an effective strategy to enhance biological performance.

## 1. Introduction

Metal complexes have attracted considerable scientific interest in recent years due to their diverse biological activities, including antimicrobial, anticancer, and antioxidant effects [1]. Among these, manganese(II) complexes coordinated with organic ligands have emerged as especially promising, owing to their distinctive physicochemical properties and versatile coordination behavior. These complexes display exceptional stability and redox characteristics, which can be precisely tuned through modifications of the ligand environment [2]. Importantly, Mn(II) complexes often demonstrate high selectivity toward specific biological targets, which can be attributed to the ability of organic ligands to dictate the geometry, electronic structure, and overall reactivity of the metal center. Such selectivity enhances their potential as therapeutic agents by reducing off-target effects and improving efficacy [1,2,3,4,5,6,7]. Moreover, the incorporation of biologically relevant ligands can facilitate interactions with biomolecules, further increasing their specificity and enabling mechanisms of action that involve oxidative stress modulation of enzyme inhibition [3]. As examples of Mn(II) complexes with organic ligands, specifically 1,10-phenanthroline, that exhibit unique chemical structures and notable biological properties, one may cite the coordination compounds synthesized and characterized by M.V. de Souza Junior et al. The complex displayed significant bactericidal activity, except against *Escherichia coli* (MIC = 7.81 μg/mL, MBC = 62.5 μg/mL), for which it demonstrated a bacteriostatic effect [4]. Two novel Mn(II) complexes were synthesized by R.K. Ahmed et al. using bisaroylhydrazone derivatives as ligands. Structural studies revealed that the metal ion is octacoordinated by two ligand molecules through bonds involving nitrogen and oxygen atoms. In addition to structural characterization, the in vitro potential of these complexes against the enzymatic activities of urease and α-glucosidase was investigated. Kinetic studies demonstrated that the compounds acted as noncompetitive inhibitors of urease, with IC_50_ values comparable to that of thiourea, a standard urease inhibitor. Results obtained from molecular docking studies were consistent with the experimental enzymatic inhibition data. Moreover, the compounds exhibited antimicrobial activity against several pathogenic bacteria, including *Bacillus cereus*, *Staphylococcus aureus*, *Escherichia coli*, and *Pseudomonas aeruginosa* [5]. The coordination compounds synthesized by M.S.S. Adam et al. using a thiophenyl imine-based ligand in the presence of divalent manganese cations resulted in the formation of two distinct mono-metallic octahedral coordination compounds with stoichiometries of 1:1 and 2:1, respectively. The biological activity of the free ligand and its corresponding coordination compounds was extensively evaluated against six pathogenic bacterial and fungal strains, as well as three human tumor and control cell lines. These investigations revealed that coordination with manganese ions significantly enhanced the inhibitory effects of the compounds compared to the free ligand, which is consistent with the chelation theory described by Tweedy. Notably, the selectivity index determined for the coordination compounds demonstrated their capacity to inhibit cancer cell proliferation more effectively than that of normal human cells, indicating promising safety profiles for potential therapeutic applications. Importantly, the incorporation of manganese ions into these coordination compounds significantly improved their biological activity, highlighting their potential as organometallic agents for anticancer and antimicrobial therapies [6].

The synthesis and comprehensive characterization of novel neutral mono-, dinuclear, and polymeric coordination compounds containing manganese(II) and derived from the nonsteroidal anti-inflammatory drug tolfenamic acid, in combination with N-donor heterocyclic ligands such as 1,10-phenanthroline, pyridine, and 2,2′-bipyridylamine, as well as O-donor ligands including water and *N*,*N*-dimethylformamide, have been reported by Psomas et al. Detailed crystallographic analyses revealed diverse coordination modes, highlighting the versatility of these compounds’ architectures. The biological evaluation of these coordination compounds demonstrated promising properties. In vitro antioxidant assays revealed that the compounds exhibit superior radical scavenging activity compared to free tolfenamic acid, with particularly notable activity against superoxide and hydroxyl radicals. The compounds also showed significant inhibitory effects on soybean lipoxygenase, suggesting potential anti-inflammatory capabilities, with the mononuclear species demonstrating the highest radical scavenging and enzyme inhibition activities. Moreover, binding studies with bovine and human serum albumins indicated strong binding affinities, with binding constants in the range of 3.56 × 10^5^ to 4.32 × 10^6^ M^−1^. Such values fall within the optimal range for effective transport and controlled release of bioactive compounds at target sites, suggesting favorable pharmacokinetic profiles. Investigations into interactions with DNA, employing UV spectroscopic titrations, viscosity measurements, and fluorescence displacement assays, revealed high DNA-binding affinities of these compounds, comparable to those of established intercalators such as ethidium bromide. These experiments support an intercalative binding mode, further corroborated by the observed ability of the coordination compounds to displace ethidium bromide from DNA. This property highlights the potential of these complexes as DNA-targeting agents [7]. Imidazo[1,2-a]pyridine and its halogen derivatives belong to a group of ligands with promising properties and potential applications in coordination chemistry. The combination of manganese(II) ions with imidazopyridines (i.e., imidazo[1,2-a]pyridine), a class of heterocyclic ligands, has emerged as a promising avenue in the pursuit of new anticancer agents. The imidazo[1,2-a]pyridine ring system was described in 1925 by Chichibabin. This system has been very poorly studied for a long time, in part because of the lack of efficient functionalization methods that would enable rapid preparation of structural variants, especially for pyridine fragments. In recent decades, a lot of work has been undertaken on the synthesis and physical properties of these compounds [8]. They can also act as inhibitors of β-amyloid formation, GABA and benzodiazepine receptor agonists and cardiotonic agents [9]. Imidazo[1,2-a]pyridine compounds have found diverse clinical applications, and various drugs containing these compounds are currently employed in the treatment of a range of diseases, including ulcers, insomnia, heart disease, and microbial infections. They have potential therapeutic effects against different cancer cell lines, including: breast, liver, colon, lung and kidney cancers. Many medicines containing imidazopyride rings in their chemical composition are used to treat various diseases, e.g., cardiac disorders, viral infection, fungal infection and bacterial [10,11].

In this paper, we report the preparation and structural characterization of three new Mn(II) complexes, supported by X-ray analysis, FT-IR and Raman spectroscopy, thermal studies, and molecular docking simulations of their potential biological activities. The studied complexes are: [Mn(3-Climpy)_2_Cl_2_(MeOH)_2_] (**1**) with 3-chloroimidazo[1,2-a]pyridine, [Mn(3-Brimpy)_2_Cl_2_(MeOH)_2_] (**2**) with 3-bromoimidazo[1,2-a]pyridine, and [Mn(impy)_2_Cl_2_]_n_ (**3**) with imidazo[1,2-a]pyridine. Notably, the double chloro-bridged manganese(II) coordination polymer (complex **3**) obtained here is relatively rare [12,13,14,15,16,17,18,19,20,21,22,23,24,25,26,27,28]. The encapsulation procedure was employed to enhance their anticancer activity in vitro. Additionally, a mechanism of action for the new Mn(II) complexes is proposed based on in vitro experimental studies, supported by molecular docking.

An important aim of this work, following the structural characterization, is to elucidate the relationship between the biological activity of the synthesized complexes and the imidazo[1,2-a]pyridine ligands, including their halogen derivatives. Although these complexes share a similar distorted octahedral coordination geometry in solution, they exhibit markedly different cytotoxic activities. These differences are attributed to variations in substituents, lipophilicity, cellular uptake, and interactions with biomolecules, highlighting the importance of both structural features and physicochemical properties in determining biological responses. We also evaluate the effect of halogen substitution in the imidazo[1,2-a]pyridine ring on the observed biological properties.

## 2. Results and Discussion

### 2.1. Syntheses

MnCl_2_·4H_2_O, imidazo[1,2-a]pyridine (*impy*), 3-chloroimidazo[1,2-a]pyridine (*3-Climpy*), 3-bromo[imidazo[1,2-a]pyridine (*3-Brimpy*) were purchased from Sigma-Aldrich (Saint Louis, MO, USA), and the solvents from Avantor (POCH S.A., Gliwice, Poland). All reagents were used without further purification.

#### Preparation of Manganese(II) Complexes **1**–**3**

MnCl_2_·4H_2_O (0.1979 g, 1.00 mmol) was dissolved in 10 mL of methanol. The synthesis was conducted at room temperature in all cases. To the stirred salt mixtures, 3-chloroimidazo[1,2-a]pyridine (0.3052 g, 2.00 mmol), 3-bromoimidazo[1,2-a]pyridine (0.3941 g, 2.00 mmol), and imidazo[1,2-a]pyridine (203 μL, 2.00 mmol) were added after being dissolved in 4 mL methanol. Immediately after their addition, white precipitates were obtained in the case of *3-Climpy* and *3-Brimpy*. The stirring continued for the next 2 h. The resulting transparent crystals of complex **3** were obtained after 1 month. The crystals of complex **3** were collected by filtration and washed with diethyl ether. The precipitates (**1** and **2**) were transferred to a filter and air-dried. After drying, the precipitates were recrystallized in methanol, and crystals were obtained 2 months after recrystallization. The crystals of complexes **1** and **2** were collected by filtration and washed with methanol and diethyl ether. The next step was the air drying of the crystals.

Anal. calcd for **1** (495.08 g/mol): C, 38.82; H, 3.66; N, 11.32 [%]. Found: C, 38.96; H, 3.44; N, 11.42 [%].

Anal. calcd for **2** (584.00 g/mol): C, 32.91; H, 3.11; N, 9.59 [%]. Found: C, 32.62; H, 2.96; N, 9.52 [%].

Anal. calcd for **3** (362.12 g/mol): C, 44.44; H, 3.34; N, 15.47 [%]. Found: C, 44.27; H, 3.33; N, 15.66 [%].

### 2.2. Crystal Structures of Complexes

**Figure 1:**
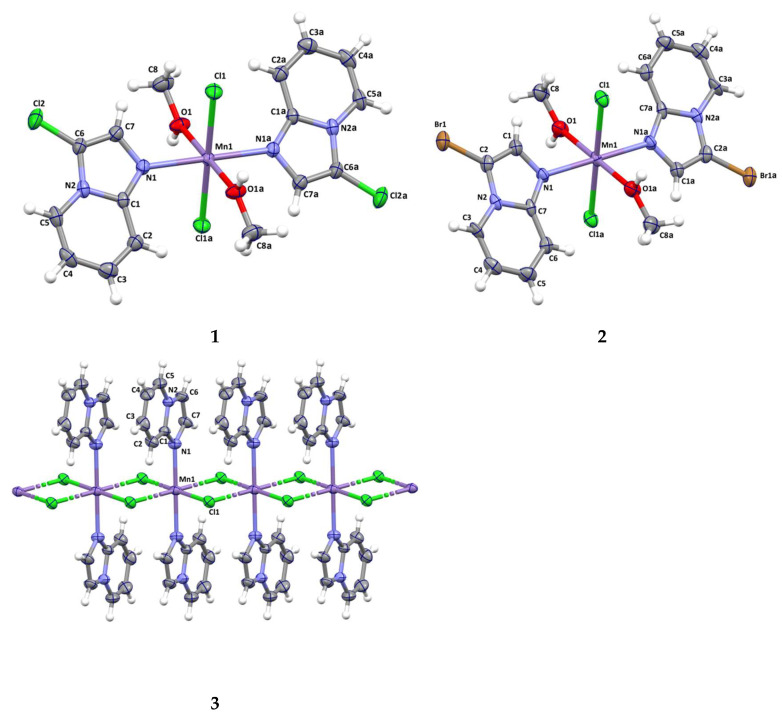
Molecular structure of **1**–**3** together with the atom numbering. Displacement ellipsoids are drawn at the 50% probability level.

Details of the crystallographic data collection, structural determination, and refinement for **1**–**3** are given in Table S1. The crystal structures have been deposited at the Cambridge Crystallographic Data Centre and allocated the deposition numbers CCDC 2,258,044 (**1**), 2,258,045 (**2**), 2,258,046 (**3**) (Figure 1).

#### 2.2.1. Structure Description of Compounds **1** and **2**

**Figure 2:**
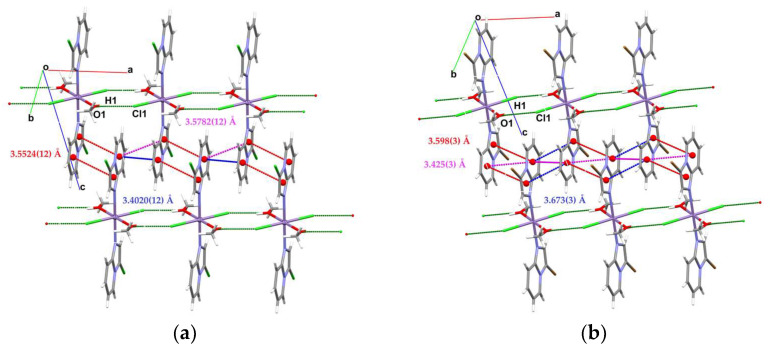
The part of molecular packing of **1** (**a**) and **2** (**b**) showing 2D supramolecular net formed via O–H···Cl hydrogen bonds and π···π stacking.

X-ray analysis revealed that the compounds [Mn(3-Climpy)_2_Cl_2_(MeOH)_2_] (**1**) and [Mn(3-Brimpy)_2_Cl_2_(MeOH)_2_] (**2**) [*3-Climpy* = 3-chloroimidazo[1,2-a]pyridine; *3-Brimpy* = 3-bromoimidazol [1,2-a]pyridine] are isomorphous and they crystallize in the triclinic space group P$\bar{1}$. The Mn(II) ions in both complexes, coordinated to two nitrogen donors from two monodentate imidazol [1,2-a]pyridine derivatives, two chloride ions and two methanol molecules, adopt a distorted octahedral geometry. Perspective views showing the molecular structure of **1** and **2** with the atom numbering are presented in Figure 1. The selected bond lengths and angles are gathered in Table S2. The *trans* angles: N–Mn(1)–N; O–Mn(1)–O and Cl–Mn(1)–Cl are linear, and *cis* angles are close to 90° [86.43 (3)–93.57 (3)° in **1**; and 86.75 (10)–93.25 (10)° in **2**]. The Mn–N bond lengths [Mn(1)–N(1) = 2.2494 (15); Mn(1)–N(1)a = 2.2494 (15) Å in **1**; Mn(1)–N(1) = 2.254 (4); Mn(1)–N(1)b = 2.254 (4) Å in **2**; (a): 1 − *x*, 1 − *y*, −*z*; (b): −*x*, 1 − *y*, 1 − *z*] are unexceptional and correlate well with distances reported for the related manganese(II) complexes. The Mn–O lengths are slightly longer than typical Mn–O lengths for six-coordinated manganese(II) complexes [28,29,30]. The elongation of the Mn–O distance can be explained by the participation of the oxygen atom in the formation of intermolecular hydrogen bond O–H···Cl. Crystal packing analysis [31] revealed that the molecules of **1** and **2** are linked through O–H···Cl-type interactions, giving rise to the formation of supramolecular chains, which are further assembled into 2D supramolecular layers via three types of weak π···π interactions (see Figure 2, Tables S4 and S5).

#### 2.2.2. Structure Description of Compound **3**

**Figure 3:**
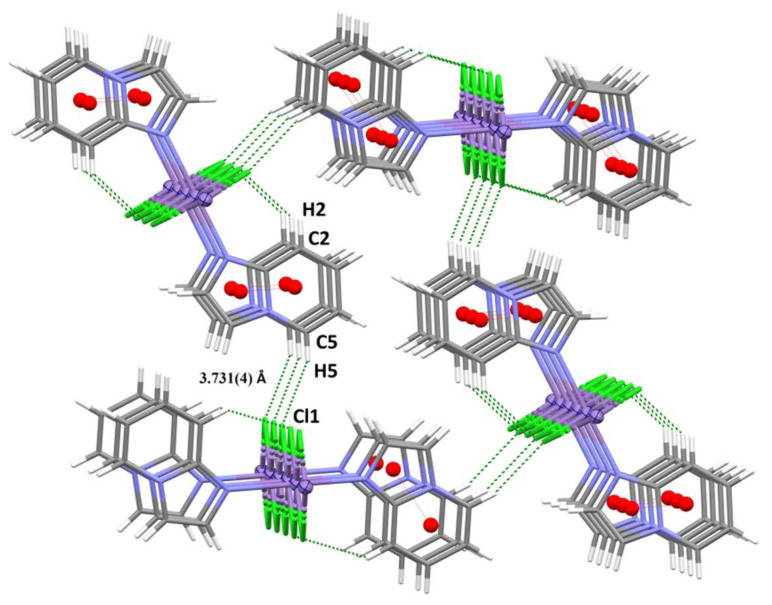
A view of the supramolecular 3D network in **3** generated by weak C–H···Cl contacts and π–π-stacking interactions.

The structure of **3** consists of uniform chains of *trans*-[Mn(impy)_2_]^2+^ units connected through double end-to-end chloro bridges that grow along the crystallographic *a* axis (Figure 3). Each Mn^2+^ ion adopts octahedral environment, defined by four chlorides [Mn(1)–Cl(1) = 2.5501 (14), Mn(1)–Cl(1)a = 2.5501 (14); Mn(1)–Cl(1)c = 2.6129 (14); Mn(1)–Cl(1)d = 2.6129 (14) Å] and two nitrogen atoms from two monodentate *impy* ligands [Mn(1) = 2.268 (5), Mn(1)–N(1)a = 2.268 (5); symmetry code: (a): 1 − *x*, 1 − *y*,−*z*; (c): −1 + *x*, *y*, *z*; (d): 2 − *x*, 1 − *y*, −*z*]. The *cis* N–Mn–N angles vary in the range of 86.77 (4)–93.23 (4)°, whereas the *trans* angles of Mn(1) are 180° due to the location of the manganese(II) ion on the special position 2a for the P2_1_/c space group. In order to estimate the angular distortion from the octahedral geometry, the SHAPE program [32] based on the Continuous Shape Measures (CShM) concept was used. The Shape value (S_Q_(P)) with respect to the octahedral geometry was found to be 0.464, while the calculated distance to the ideal trigonal prism was 16.433. The intrachain Mn···Mn separation was 3.752 (4) Å, a value which was much shorter than the shortest interchain metal–metal distance [9.820 (1) Å for Mn(1)···Mn(1)g; (g) = 1 − *x*, −½ + *y*, ½ − *z*]. The linear chains of **3** were linked through π···π [Cg(5)···Cg(6)^e^ = 3.731 (4) Å; (e): 1 + *x*, *y*, *z*] stacking, leading to supramolecular 2D motifs. These layers were further associated via very weak C(5)–H(5)···Cl(1)- and C(2)–H(2)···Cl(1)-type interactions forming supramolecular 3D structures (Figure 3; Tables S4 and S5). Notably, double chloro-bridged manganese(II) coordination polymers are relatively rare. A search in the Cambridge Structure Database (Version 5.37) showed only 17 such systems [12,13,14,15,16,17,18,19,20,21,22,23,24,25,26,27] (see Table S3).

### 2.3. Thermal Analysis

The suggested decomposition products of **1**–**3** in the solid state are based on mass loss calculations. Thermogravimetric, differential thermal analysis (DTG) curves and corresponding data are illustrated in Figure S1 and in Table S6.

Complex **1** decomposes in four endothermic processes, the first step at 234 °C and the second at 334 °C, corresponding to the loss of 2 chloride molecules (2 atoms from the coordination sphere), 2 water molecules and 2 nitrogen molecules, respectively. The assignment of these two steps involving the loss of the imidazo[1,2-a]pyridine ring from the *3-Climpy* ligand is in the next two steps (endothermic peaks at 381 °C, 605 °C). We suggest a residual product, which can be MnO_2_, for thermal decomposition of **1**. Complex **2** begins to decompose at 50 °C. The decomposition stage between 50 and 145 °C with an endothermic effect at 83 °C is related to the removal of the two water molecules with a mass loss of 5.2% (*calc.* 6.2%). The next stages of **2** result in the loss of Br_2_ molecules at 229 °C and Cl_2_ molecules at 383 °C, found to be 29.6% (calc. 27.4%) and 10.6% (*calc.* 12.1%), respectively. The two endothermic peaks at 362 °C and 576 °C in the TGA curve of **2** suggest decomposition of the ligand ring of *3-Brimpy*, a mass loss of 40.7% (*calc*. 39.4%). The obtained residue product is MnO_2_ as well.

The TG curve for **3** shows that the complex is stable up to 50 °C, with the first weight loss of 21.0% (*calc.* 19.6%) at 210 °C corresponding to the loss of chloride molecules from the coordination bridge. In the next stage, the endothermic effect at 290 °C is related to removal of the two N_2_ molecules. In the next two stages, with endothermic peaks at 416 °C and 650 °C, the found sum of mass loss is 35.6% due to a release of imidazo[1,2-a]pyridine ring fragments. The successive mass loss (73.9%, *calc.* 76.0%) may lead to MnO_2_ formation.

### 2.4. Spectroscopic Studies

The FT-IR and Raman spectra of **1**–**3** in the solid state in the middle and far infrared regions are shown in Figures S2–S4, respectively. A vibrational assignment of the experimental spectra was performed based on the calculated frequencies (B3LYP-D3/def2-TZVP) and analysis of the mode displacements using GaussView 6 and Chemcraft programs (https://www.chemcraftprog.com/, access: 12 March 2026). The characteristic medium bands at 3336 and 3339 cm^−1^ in the FT-IR spectra of **1** and **2** can be assigned to the ν (O-H) stretching vibration involved in the formation of intermolecular hydrogen bonds O-H···Cl, respectively. The stretching vibration ν (C-H) of *3-Climpy*, *3-Brimpy* and *impy* ligands in Mn(II) complexes generates weak bands in the 3154–2839 cm^−1^ spectral region of the FT-IR spectra (Raman: 3155–2842 cm^−1^). The imidazo[1,2-a]pyridine ring stretching vibrations (ν (C-C) and ν (C-N)) generate, in the FT-IR spectra of the discussed Mn(II) complexes, a few strong or medium bands, e.g., at 1634 cm^−1^ (m), 1498 cm^−1^ (s), 1364 cm^−1^ (m), and 1305 cm^−1^ (s) for **1** (Raman: 1636 cm^−1^ (m), 1499 cm^−1^ (vs), 1450 cm^−1^ (m), 1344 cm^−1^ (m), and 1395 cm^−1^ (vs)). The strong or medium bands at 1239 cm^−1^ (s) and 1143cm^−1^ (s), 1229cm^−1^ (s) and 1143cm^−1^ (s), and 1237 cm^−1^ (m) and 1144 cm^−1^ (s) in the FT-IR spectra of **1**–**3** originate from δ (C-H) (in the Raman spectra, these bands are medium as well). In the FT-IR spectra of ligands and complexes **1**–**3**, characteristic bands with strong intensities at 749/735 cm^−1^, 749/735 cm^−1^, and 734/720 cm^−1^ are observed. These peaks are due to out-of-plane bending vibrations γ (C-H) and torsion of *impy* ring fragments from ligands, respectively.

From a coordination chemistry point of view, the most informative diagnostic region confirming the structure and coordination mode is the far-infrared range; therefore, we list and assign the most important bands in the vibrational spectra within this region. The contribution of the ν (Mn-O) stretching vibration in complexes **1** and **2** to the ring bending vibration can be found in the medium band at 500 cm^−1^ and 488 cm^−1^ in the FT-IR spectrum, respectively.

The medium band in the FT-IR spectra at 422 cm^−1^ (**1** and **2**, Raman: 422 cm^−1^, 428cm^−1^, respectively) and at 419 cm^−1^ (**3**, Raman: 422 cm^−1^) can be assigned to the torsion of the τ R_6_ ring (imidazole) from *3-Climpy*, *3-Brimpy* and *impy*. As Nakamoto suggests [33], in the FT-IR spectrum of polymeric octahedral complex **3**, we can expect two ν (Mn-Cl) and one ν (Mn-N) stretching vibrations. In our case, we observe these two stretching ν (Mn-Cl) bands at 430 cm^−1^ and 419 cm^−1^, and they are coupled with bending δR_6_ and torsion τR_6_ vibrations of the *impy* ring, respectively. In complexes **1** and **2**, this stretching (ν Mn-Cl) is observed as one band at 422 cm^−1^ in the FT-IR spectrum of these complexes. In addition, the bending δ (Cl-Mn-Cl) vibrations generate a medium band at 159 cm^−1^ in the FT-IR spectrum of **3** (Raman: overlapped), at 181 cm^−1^ in **1**, and 157 cm^−1^ in **2**. The stretching ν (C-Br) vibrations in ligand *3-Brimpy* generate a medium peak at 351 cm^−1^ in FT-IR and 349 cm^−1^ in the Raman spectrum of **2**.

The medium bands at 256 cm^−1^ (weak in Raman) in the FT-IR spectrum of **3** can be assigned to the stretching ν (Mn-N) vibrations, and in the FT-IR spectra of **1** and **2**, this band is overlapped. Moreover the characteristic torsion of two rings (pyridine and imidazole) of the ligands relative to each other generates the band at 201 cm^−1^ (Raman: 203 cm^−1^), known as butterfly in the FT-IR spectrum of **3**.

### 2.5. Mn(II) Complexes in MeOH and DMEM Solution by Spectroscopic Study

Firstly, the stability of the solid-state synthesized complexes **1**–**3** was evaluated by thermal analysis. To investigate their behavior in solution, UV-Vis spectra were recorded in DMEM (Dulbecco’s Modified Eagle Medium) (Figure S5a). In DMEM, absorption in the 200–300 nm region showed pronounced saturation effects (Figure S5a), which is consistent with the strong intrinsic background absorption of this medium and limits reliable spectral analysis even at relatively low concentrations. Therefore, UV–Vis measurements were additionally performed in methanol, which was selected due to solubility limitations in other solvents. The corresponding spectra are presented in Figure S5b.

Under these conditions, the 200–300 nm region could be properly evaluated. The UV–Vis spectra of complexes 1–3 remained essentially unchanged over time, both in the positions and intensities of the π → π* transitions (210–230 nm) and the ligand-based n → π*/charge-transfer bands (280–300 nm). A slight saturation around 300 nm was observed, indicating that this transition reaches maximal absorbance at an early stage.

Additionally, FT-IR spectra were collected after dissolving complexes **1**–**3** in methanol (Figure S6). Methanol was used for FT-IR measurements because spectra recorded in DMEM contained numerous additional bands originating from medium components, such as amino acids, proteins, and inorganic salts, which significantly interfered with clear identification and interpretation of the vibrational features of the investigated complexes.

Assessing the stability of manganese complexes is inherently challenging, as they readily undergo redox transformations depending on solvent, light, and other environmental factors. Time-dependent UV-Vis spectra collected for complexes **1**–**3** in DMEM over 72 h did not show significant spectral changes. The recorded spectra remained essentially unchanged throughout the monitoring period, suggesting that the predominant form present immediately after dissolution persists in solution for at least 72 h under the applied experimental conditions. Nevertheless, such spectroscopic observations do not allow for an unequivocal determination of complete solution stability.

More detailed insight into the processes occurring within the coordination sphere of the Mn^2+^ center was obtained from FT-IR analysis. For complexes **1** and **2**, the ν(O–H) stretching bands at 3339 and 3336 cm^−1^ disappeared after dissolution in methanol, indicating the loss of two coordinated methanol molecules. Importantly, the remaining vibrational features, particularly ν(Mn–Cl) (422 cm^−1^) and δ(Cl–Mn–Cl) (181 cm^−1^ for **1** and 157 cm^−1^ for **2**), remained unchanged. Complex **3** exhibited identical FT-IR spectra in the solid state and after dissolution in methanol, retaining ν(Mn–Cl) (430 and 419 cm^−1^), δ(Cl–Mn–Cl) (159 cm^−1^), and all characteristic ligand vibrations.

The spectroscopic data suggest that, in solution, complexes **1** and **2** may undergo structural rearrangement. In particular, the formation of chloro-bridged manganese(II) species cannot be excluded. The observed spectral similarities indicate that, upon dissolution, complexes **1** and **2** may adopt structures consistent with μ-Cl-bridged manganese(II) coordination polymers analogous to that identified in complex **3**, while remaining reasonably stable under the examined conditions.

In addition, the stabilizing effect of the biological medium likely arises from interactions with amino acids, proteins, phosphates, or metal ions present in the medium, which may buffer redox activity or stabilize Mn(II) through weak associative interactions. On this basis, the structures identified by spectroscopic analysis were adopted as models for molecular docking studies. Considering this, we selected the [Mn(3-Ximpy)_2_Cl_4_]^2−^ model for the computational analysis as it captures the essential features of the μ-Cl-bridged coordination environment indicated by the solution data. We have revised the discussion to clarify this choice and to emphasize that the calculations aim to represent the solution behavior of complexes **1** and **2** rather than to definitively prove the existence of a fully discrete [Mn(3-Ximpy)_2_Cl_4_]^2−^ species.

### 2.6. Biological Activities

#### 2.6.1. Primary Screening of Antiproliferative Activity

Primary screening of antiproliferative activity of the manganese complexes (**1**, **2** and **3**) was performed by the commonly used MTT assay on four human cancer cell lines: HT-29 (colorectal adenocarcinoma), MCF-7 (breast), A549 (lung adenocarcinoma), DU145 (prostate) and PANC-1 (pancreatic). In addition, normal human keratinocyte (HaCaT) was used as control cell lines to assess the toxicity of these complexes control. The results are summarized in Table 1.

The IC_50_ values examined for compounds are compared to those obtained for clinically used cisplatin (CDDP) under the same experimental conditions. In general, all investigated Mn(II) compounds demonstrated low cytotoxicity towards cancer and normal cell lines except PANC-1 cancer cells. Pancreatic cancer remains one of the most lethal malignancies due to its immunosuppressive microenvironment and limited therapeutic options, highlighting the urgent need for innovative multimodal treatment approaches [34,35]. Taking account of the above, unexpectedly, the activity of all Mn complexes against pancreatic cell lines was higher than for a well-known anticancer drug—cisplatin. The highest activity was shown by **2**, which differs from **1** and **3** by the presence of a bromide substituent in the imidazo[1,2-a]pyridine ring, while **1** contains chloride and **3** does not. It was proven, in different studies, that brominated pyridine derivatives can enhance lipophilicity and membrane permeability, which correlates with stronger biological activity, including cytotoxic effects [36,37,38].

**Figure 4:**
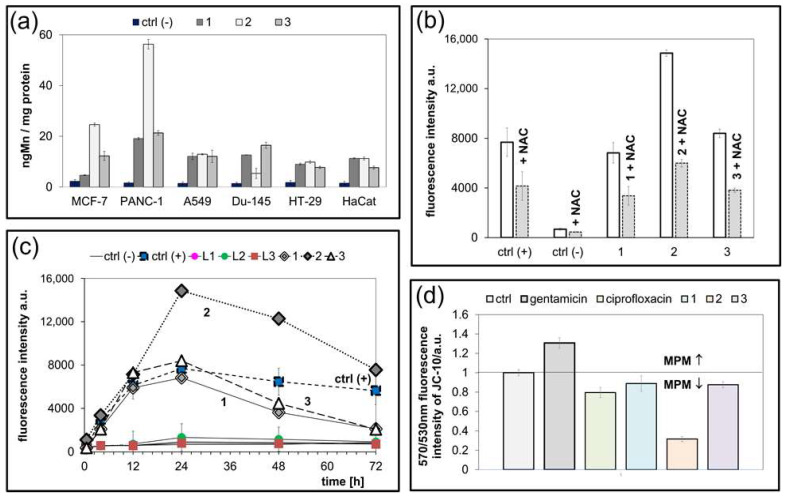
(**a**) Cellular uptake. Final intracellular manganese concentration expressed as ng Mn per mg protein after 24h incubation with the A549, MCF-7, PANC-1, DU-145, HT-29, and HaCat cell lines for complexes **1**, **2**, **3** in c = 1 μM. (**b**) The ROS production in PANC-1 cells after 24 h using H_2_DCF-DA for Mn compounds (**1**, **2**, **3**) with and without NAC (5 mM); ctrl (+): H_2_O_2_ as positive control, and ctrl (−): negative control, cells without compound. (**c**) The ROS generation was monitored by H_2_DCF-DA assay in PANC-1 cells during steady incubation with Mn complexes for 30 min, 6, 12, 24, 48 and 72 h in c = 1 μM; ctrl (+): H_2_O_2_ as positive control, and ctrl (−): negative control, cell without compound. (**d**) Alteration in mitochondrial membrane potential (MMP) is given as an emission ratio 570 nm/530 nm (ctrl—untreated cells; ciprofloxacin—a negative control; gentamicin—a positive control).

ICP-MS was performed to assess whether the Mn(II) uptake correlates with anticancer in vitro activity of complexes. As shown in Figure 4a, manganese ion accumulation was observed to be most pronounced in PANC-1 cells treated with **2**, compared to all other discussed Mn(II) complexes. Interestingly, we observed that **2** is efficiently accumulated only in PANC-1 cells, leading to the conclusion that its high cytotoxicity is connected with intracellular accumulation. Complexes **1** and **3** did not show such a potent anticancer in vitro activity, presumably because their cellular accumulation was lower. Moreover, the accumulation of all complexes varied significantly between cancerous and normal cells, with lower accumulation observed in the HaCaT cell lines. These findings strongly support the observed differences in cytotoxicity between the compounds in cancer cell (PANC-1) lines and the normal cell lines (Figure 4a).

Since manganese complexes are well-known to be highly redox active [39,40,41], intracellular reactive oxygen species (ROS) generation, related to the treatment with manganese complexes (**1**, **2**, **3**), was evaluated in PANC-1 cancer cells (Figure 4b,c). ROS generation was determined by using a fluorescent H_2_DCF-DA ROS probe (λ_ex_ = 495 nm, λ_em_ = 530 nm). Hydrogen peroxide was used as a positive control, with N-acetyl derivative of the amino acid L-cysteine (NAC) (Figure 4b) as a scavenger. The scavenging activity of the latter is enabled by its free thiol (-SH) groups. Additionally, NAC served as a precursor for the synthesis of reduced glutathione (GSH), a critical intracellular antioxidant. Numerous studies have demonstrated that depletion of GSH is closely associated with ROS-mediated mechanisms of drug-induced apoptosis. NAC is frequently employed in experimental models to validate the involvement of oxidative stress in these apoptotic pathways [42,43,44].

All Mn(II) complexes were able to generate ROS. Complex **2** was the most active among all studied compounds, including the positive control H_2_O_2_. The order of the ROS levels induced by the studied complexes in PANC-1 cancer cells can be presented following **2** > **3** ≥ **1**. Moreover, treatment with NAC resulted in a significant reduction in ROS production. This data confirms the oxidative effect of the Mn(II) compounds, especially **2**, in PANC-1 cancer cells and its important role in the generation of cellular stress. Notably, the cells treated with complexes in the absence of NAC can be considered as capable of increasing the ROS generation even up to 100% when compared with untreated control cells. As shown in Figure 4c, the intracellular ROS concentration increases within 24 h of incubation and then significantly decreases over time. This observation can be linked to cellular lipid peroxidation, as has been presented before in many papers [45,46].

Reactive oxygen species (ROS) and mitochondrial membrane potential (ΔΨm) are closely interdependent: ROS generation is largely driven by the electrochemical gradient across the mitochondrial membrane, while excessive ROS can, in turn, disrupt ΔΨm. In cancer cells, this dynamic is tightly regulated, making mitochondria an attractive target for therapeutic intervention using redox-active metal complexes [47]. To evaluate the role of mitochondrial dysfunction in ROS-mediated cytotoxicity induced by bimetallic complexes, changes in ΔΨm were assessed using the JC-10 fluorescent probe (Figure 4d). As controls, gentamicin, known to increase ΔΨm, and ciprofloxacin, which decreases ΔΨm, were employed to validate the assay system. Manganese complexes reduced the mitochondrial membrane potential (MMP) compared to the untreated cells. What is most important is that complex **2** caused the most significant decrease in MMP, leading to the conclusion that mode of action in Mn(II) complexes with bromopyridine derivatives can relate to mitochondrial damage together with ROS production. Complexes **1** and **3** do not decrease MMP significantly, but this fact can be related to their low permeability and inefficient uptake. A reduction in the mitochondrial membrane potential (ΔΨm) can initiate the release of cytochrome c from the mitochondrial intermembrane space into the cytosol, particularly under conditions where the pro-apoptotic protein Bax is overexpressed. Once in the cytoplasm, cytochrome c associates with Apaf-1 and procaspase-9 to form the apoptosome, thereby activating the caspase cascade and promoting apoptosis. Conversely, the anti-apoptotic protein Bcl-2 can bind to Bax, forming heterodimers that inhibit Bax oligomerization and pore formation in the mitochondrial outer membrane. This interaction effectively suppresses cytochrome c release and delays or prevents the initiation of apoptotic signaling [48,49].

#### 2.6.2. Polymeric Micelle-Mediated Delivery of Mn(II) Complexes

Effective uptake of any metallodrug, involving metal compounds, is a crucial factor for selective and efficient chemotherapies. The internalization concerns both cancer and normal cells since the uncontrolled destruction of normal cells is mostly associated with the high systemic cytotoxicity. The development of multidrug resistance can also be linked with too high uptake of chemotherapeutics by cells. Moreover, since most metal complexes are not sufficiently soluble in water, their bioavailability can be increased by encapsulating them in appropriate carriers. These serious concerns support the need to look for novel safe targeted treatments based on nanotechnology. Thus, to face this challenge, Pluronic P-123 (PEO20-PPO70-PEO20) with long hydrophobic blocks was chosen to prepare micelles with encapsulated Mn(II) complexes. This polymer is commercially available, and its biocompatibility and safety have been proven by the FDA [50].

Two Mn(II) complexes, **1** and **3**, were selected to be encapsulated into polymeric micelles, denoted as **1_M** and **3_M**, respectively, because of their lower uptake efficiency compared to compound **2**. Drug loading content (LC), encapsulation efficiency (EE), and hydrodynamic diameter and Zeta potential, summarized in Table 2, were determined with application of ICP-MS and DLS techniques, respectively.

Both studied complexes were characterized by effective loading, as proven by high values of LC and EE. This suggests an efficient delivery of compounds to cells, along with their mean micelle size (**1_M**, **3_M**), which is lower than 50 nm, the critical size for avoiding capture by the reticuloendothelial system (RES). Negative values of Zeta potentials of stable **1_M** and **3_M** resulted from a slightly negative potential of uncharged PEO amphiphilic copolymers.

Then, the cytotoxicity of two polymeric formulations **1_M** and **3_M** was studied in the selected cell lines: human pancreatic/duct carcinoma (PANC-1) and human keratinocytes (HaCaT) as a reference normal somatic line. The cell viability was assessed by MTT assay after 24h incubation with the studied formulations and an additional 24 h for recovery time in a formulation-free media (Table 3).

**Figure 5:**
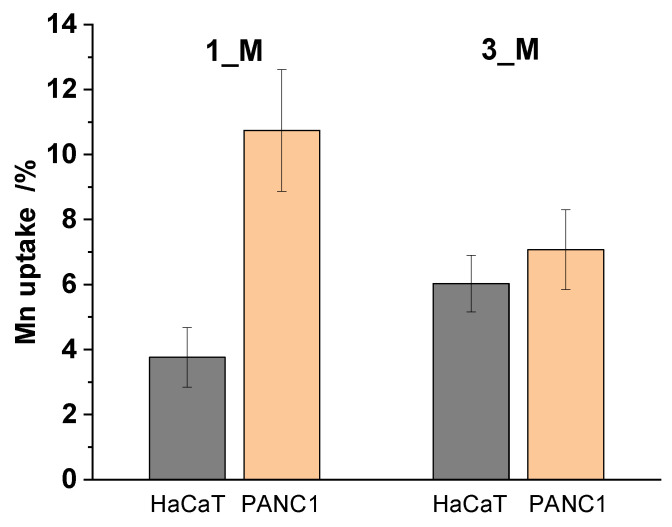
Cellular uptake. Final intracellular manganese concentration expressed in percentages after 24 h incubation of **1_M** and **3_M** (c = 0.05 mg/mL and 0.03 mg/mL, respectively) with the PANC-1 and HaCat cell lines.

As shown in Table 3, both **1_M** and **3_M** nanoformulations exhibited cytotoxicity towards the studied human cancer cells when compared with the previously studied **1** and **3** compounds (vide supra, Table 1). Notably, the Pluronic P-123 micelles with encapsulated compounds **1** and **3** exhibited IC_50_ values one order of magnitude higher, which proves the selectivity of the resulting formulations. Finally, cellular uptake of **1_M** and **3_M** formulations was determined by ICP-MS to support an enhanced pattern of their cellular uptake into PANC1 over HaCaT cells and when compared with the corresponding complexes **1** and **3** (Figure 5).

Interestingly, although the accumulation of **1_M** formulation in PANC1 cells was significantly higher than that of **3_M** formulation, the cytotoxicity of **3_M** towards these cells was definitely greater (Figure 5, Table 3). It is known that Pluronic P-123 nanoformulations tend to aggregate spontaneously, releasing their load in relation to pH [50]. Thus, it can be supposed that in a pathological tumor microenvironment characterized by lower pH (e.g., pH = 5.5 for endosome, pH = 5.0 for lysosome), in comparison with normal tissues (pH = 7.4), efficient release of Mn(II) complexes will be facilitated.

All these above-described findings allowed us to conclude that the encapsulation of compounds **1** and **3** into polymeric micelles enhanced their cellular uptake and activity. The successful and efficient uptake of poorly soluble Mn(II) complexes occurred mainly because of the physicochemical characteristics of Pluronic P-123, which is capable of interacting with cell membranes, leading to decreased microviscosity and pore formation, resulting in the acceleration of internalization.

#### 2.6.3. Interactions with Proteins and DNA—Experimental Studies and Molecular Docking

**Figure 6:**
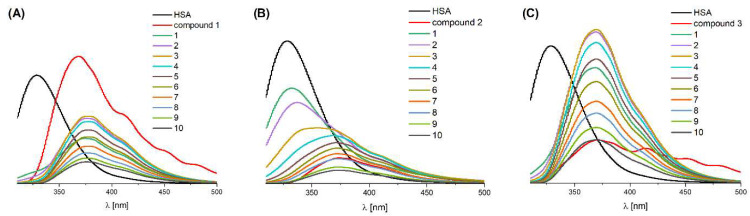
Quenching of HSA fluorescence (C = 5 × 10^−5^ M) by compound (**A**) **1**, (**B**) **2** and (**C**) **3** in 20% DMSO (1:1–10 HSA:compound molar ratio in 10 mM HEPES buffer at pH 7.4).

Proteins, such as the abundant human serum albumin (HSA), play key roles in biochemical reactions and molecular transport, making their interactions with potential drugs essential for understanding chemical and biological processes in the human body [51,52,53]. We decided to compare the experimental results with molecular docking in order to better discuss the mechanism of action of complexes **1**–**3**. The tertiary structure of HSA contains the homologous domains I, II and III with subdomains A and B. HSA has only one tryptophan residue (Trp-214) in its structure, located in subdomain IIA [52]. Drug interactions with albumin can be monitored by Trp-214 emission quenching. Interactions of Mn(II) complexes (as potential drugs) with HSA are a common subject of studies in the literature [54,55]. The interaction of Mn(II) complexes with human serum albumin (HSA) was studied by a tryptophan emission quenching experiment. In all the studied cases, after the addition of the compound to HSA, the formation of a new band located at higher wavelengths was observed (Figure 6).

In detail, a sharp decrease in the intensity of the HSA band at 329 nm was observed after titration of the protein with complexes **1** and **3** (Figure 6A,C). In the case of the addition of complex **2** to HSA, a gradual decrease in intensity at λ_max_ = 329 nm is visible (Figure 6B). The maximum of this band is located at ~375 nm (with a shoulder at ~412 nm) and additionally overlaps with the emissions of the compounds themselves. During titration with the complexes (in the case of **1** and **3**), the intensity of this band initially increases; however, it starts to decrease when the HSA:compound molar ratio is 1:4, which suggests an interaction of Mn(II) complexes with HSA (Figure 6A,C), while in the case of **2**, only a simple decrease in the intensity of the HSA–compound **2** band was observed (Figure 6B).

**Figure 7:**
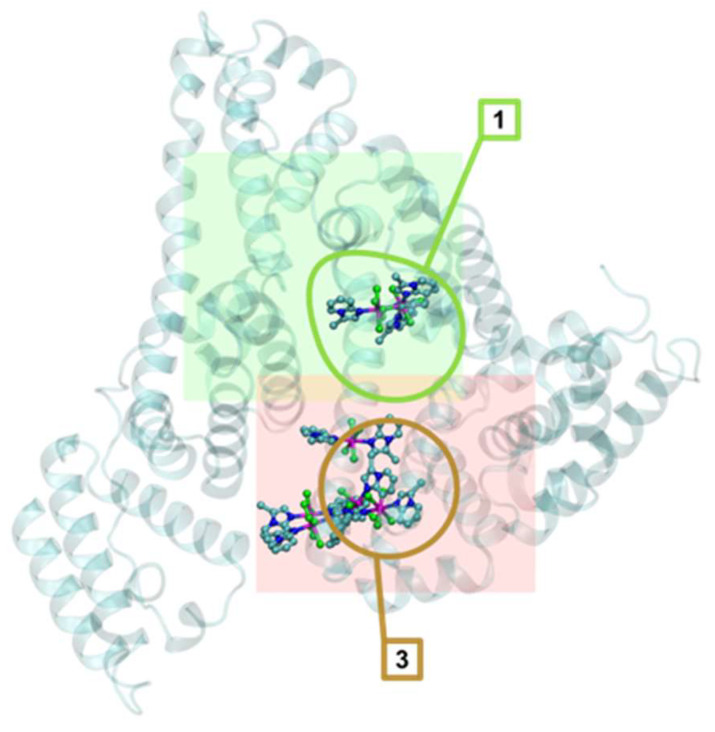
Representative binding poses of **2** to the sites of human serum albumin. The protein structure is presented as a transparent cyan backbone; the ligands are represented as ball-and-stick models. Elements color-coded: C—cyan; N—deep blue; O—red; P—golden; Cl—green; Mn—purple.

To gain deeper insight into these experimentally observed interactions, we complemented the fluorescence quenching studies with molecular docking analysis. The scores of all representative binding poses interacting with HSA are presented in Table S7, and the binding of different compounds is visualized in Figure 7 for **2**, and Figures S7 and S8 for **1** and **3**, respectively. The HSA binding scores are noticeably better than for the interaction with DNA. In all cases, only sites 1 (next to the Trp-214 residue) and 3 (the heme-binding pocket) were occupied, but without significant differences in binding affinity. The results may suggest that **2** shows the highest affinity to HSA (Figure 7), but the difference between **2** and **3** is only 1.7 kcal/mol, which is about half of the standard error of the AutoDock4 force field [56]. The differences in affinity between binding sites are below kT in all cases, corresponding to estimated dissociation constants (Kd) between 15 and 250 µM.

**Figure 8:**
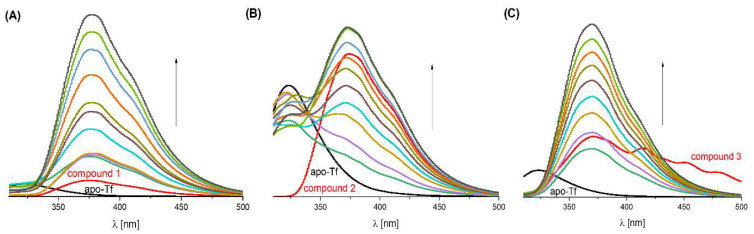
Interaction of apo-Tf (C = 3.6 × 10^−6^ M) with compounds (**A**) **1**, (**B**) **2** and (**C**) **3** in 20% DMSO (1:1–10 apo-Tf:compound molar ratios in 10 mM HEPES buffer at pH 7.4).

Serum transferrin (Tf), which transports Fe(III) ions and exists partly as apo-Tf in blood, has been shown to interact with all Mn(II) complexes, highlighting its relevance in designing new therapeutic agents [57,58,59]. The intensity of the emission band for apo-transferrin (apo-Tf) at 324 nm changes after titration with the tested compounds (Figure 8). However, this intensity does not change in a regular manner. Similarly to the case of HSA, after adding the compound, a new band is created at ~375 nm, and it overlaps with the emissions of the tested compounds (Figure 8).

With the increase in the molar ratio of the tested compounds, the intensity of this band gradually increases, which may indicate that the tested Mn(II) complexes bind to apo-Tf and change the protein structure (Figure 8).

To further investigate these observations, molecular docking was performed. The results of all representative binding poses with apo-Tf are presented in Table S8.

**Figure 9:**
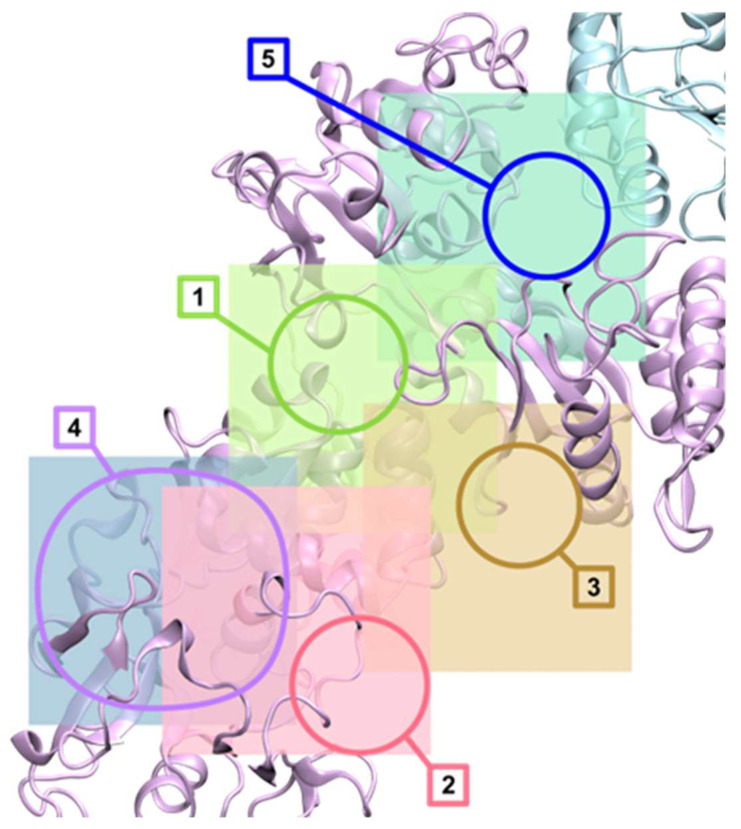
Positions of binding sites 1–5 on apo-Tf chain A. Docking grids represented by transparent boxes, the protein represented as a backbone secondary structure.

**Figure 10:**
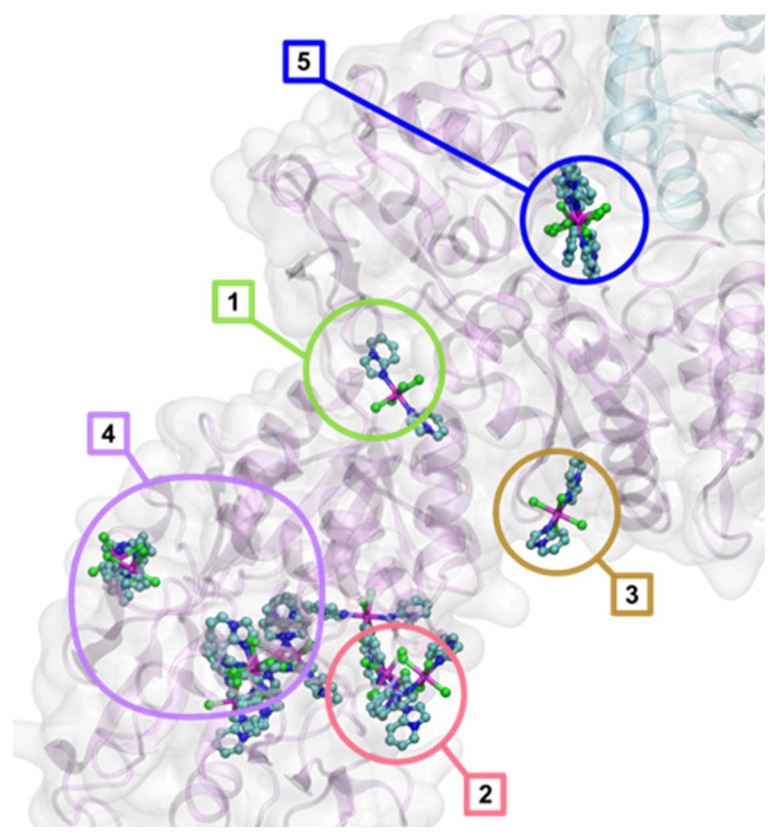
Representative binding poses of **3** at the sites of human serum apo-transferrin. The protein structure is shown as a transparent gray surface with a colored backbone: chain A is in magenta, and chain B is in cyan. The ligand poses are depicted as ball-and-stick models, with atom colors as shown in Figure 7.

Analysis showed that the preferred binding site for all studied complexes is the space between the protein chains, designated as site 5 (Figure 9), although for **3** (Figure 10) sites, 1, 2, and 4 were also occupied within kT of the best result for site 5. All predicted binding sites were sampled, with site 3 consistently yielding the worst results. For all compounds (Figure 10, Figures S9 and S10), their affinity for apo-Tf was found to be weaker than for HSA, but still within the standard error range of the AutoDock4 force field.

**Figure 11:**
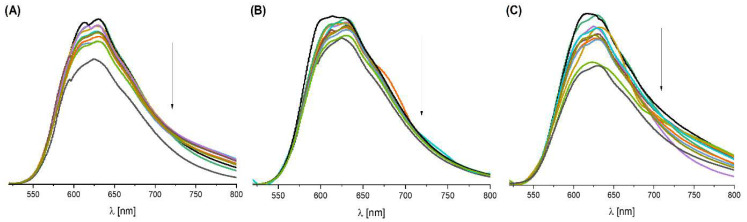
Quenching of EB-CT-DNA fluorescence (C = 5 × 10^−5^ M) by compound (**A**) **1**, (**B**) **2** and (**C**) **3** in 20% DMSO (1:1–10 EB-CT-DNA:compound molar ratios in 10 mM HEPES buffer at pH 7.4).

Furthermore, the interactions with DNA were investigated by studying the binding modes of the Mn(II) compounds to CT-DNA. The molecules can bind to CT-DNA in two ways: covalently or non-covalently. Covalent interactions are irreversible and are mainly represented by inter- and intrastrand cross-links. In turn, major or minor groove binding, intercalation, and electrostatic interactions are classified as non-covalent, reversible interactions [60]. It is mentioned in the literature that Mn(II) complexes often interact with DNA via intercalation [61,62]; therefore, we aimed to determine whether this non-covalent type of binding occurs for the complexes studied. To assess the ability of the complexes to bind to CT-DNA, we used ethidium bromide (EB), a typical intercalator. If the tested compound interacts with CT-DNA more strongly than the dye (EB) (λ_ex_ = 540 nm), then the intensity of the emission band for EB-CT-DNA at 625 nm decreases. A slight decrease in the intensity of the EB-CT-DNA band at 625 nm can be seen after the addition of the compounds (Figure 11). These small changes in the band intensity suggest a weak interaction of the tested Mn(II) compounds with CT-DNA.

The weak interactions of Mn(II) complexes with CT-DNA suggest that the tested compounds should not damage DNA. This fact was confirmed by a gel electrophoresis experiment with plasmid DNA. DNA was not damaged regardless of the concentration of the tested compounds (Figure S7). In the control sample (ctrl), the native form of DNA (form I), as well as the low intensity of the band of the circular form of DNA (form II), is visible (Figure S11, lane 1). Furthermore, the addition of the tested compounds to plasmid DNA did not affect the changes in the DNA structure at all (Figure S11, lanes 2–10).

**Figure 12:**
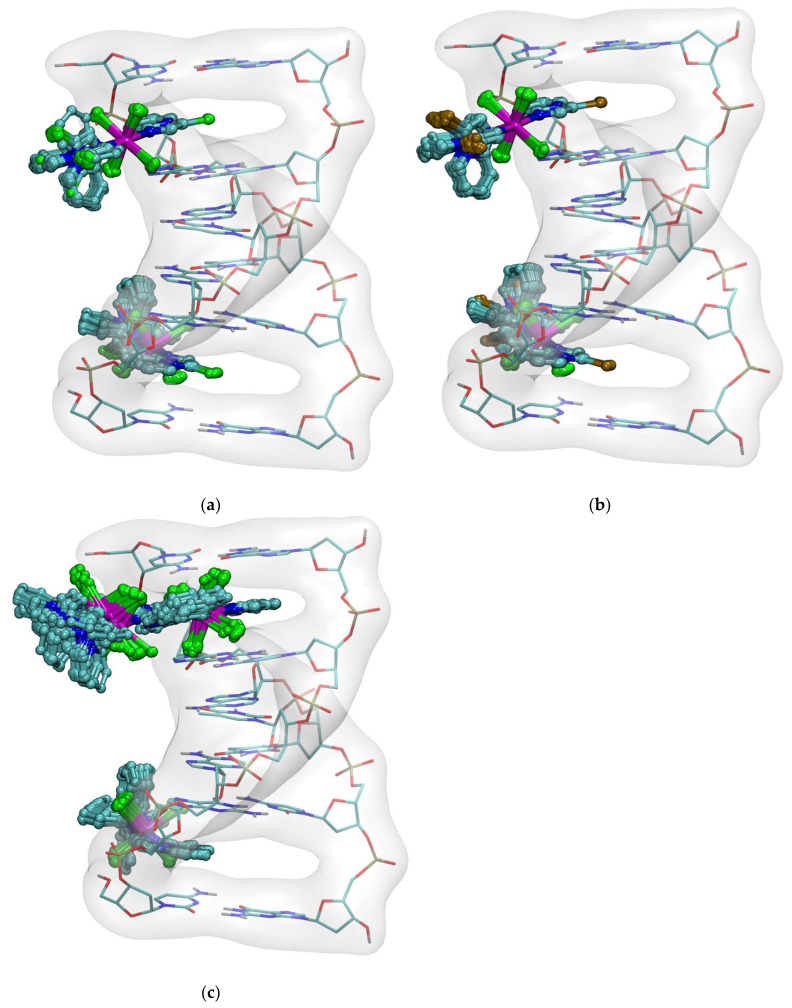
Binding poses of **1** (**a**), **2** (**b**) and **3** (**c**) docked to the DNA crystal structure 1Z3F. The element colors are described in Figure 7.

Molecular docking was conducted to further elucidate the binding modes. Representative binding poses of the studied compounds with the DNA B-form double helix are shown in Figure 12, and the quantitative results are summarized in Table 4.

**Figure 13:**
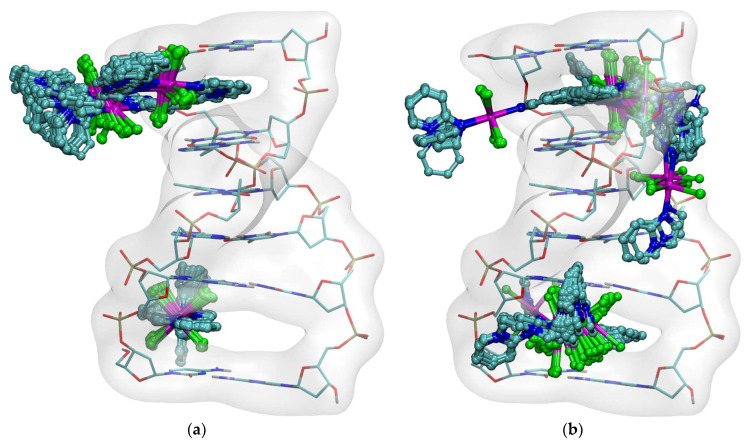
Results of docking of **3** to the DNA molecule from PDB structure 1Z3F. (**a**) using RESP atomic charges, and (**b**) using Gasteiger charges as calculated by AutoDock Tools. The ligands shown in ball-and-stick representation (all 300 calculated possess), the DNA presented as thin sticks with transparent gray molecular surface. Elements color-coded: C—cyan; N—deep blue; O—red; P—golden; Cl—green; Mn—purple.

Docking revealed that the only observed binding mode for all three manganese compounds involved intercalation of a single aromatic ring. Importantly, **3** (Figure 13) was repelled, and even for **2**, the interaction energy was comparable to kT. These results indicate that the binding is too weak to be significant, as such a negligible affinity would be insufficient to disrupt the π–π stacking interactions between DNA bases.

## 3. Materials and Methods

### 3.1. Experimental Structural Characterization

#### 3.1.1. X-Ray Analysis

Single crystal X-ray diffraction data of **1**–**3** were collected on a Gemini (New York, NY, USA) A Ultra diffractometer equipped with Atlas CCD detector and graphite monochromated Mo*K*α radiation (α = 0.71073 Å) at room temperature. Unit cell determination and data integration were carried out using the CrysAlis (Sauget, IL, USA) package of Oxford Diffraction [63]. The structures were solved by direct methods using SHELXS and refined by full-matrix least-squares analysis on F^2^ using SHELXL-2014 [64]. All non-hydrogen atoms were refined anisotropically. The hydrogen atoms were placed in calculated positions refined using idealized geometries (riding model) and assigned fixed isotropic displacement parameters, *d*(C–H) = 0.93 Å, *U*_iso_(H) = 1.2 *U*_eq_(C) (for aromatic); and *d*(C–H) = 0.96 Å, *U*_iso_(H) = 1.5 *U*_eq_(C) (for water). Details of the crystallographic data collection, structural determination, and refinement for **1**–**3** are given in Table S1. The crystal structures have been deposited at the Cambridge Crystallographic Data Centre and allocated the deposition numbers CCDC 2,258,044 (**1**), 2,258,045 (**2**), and 2,258,046 (**3**).

#### 3.1.2. Vibrational Spectroscopy

ATR FT-IR spectra of Mn(II) complexes, imidazo[1,2-a]pyridine, 3-chloro[imidazo[1,2-a]pyridine and 3-bromo[imidazo[1,2-a]pyridine were collected using a Bruker (Ettlingen, Germany) Vertex 70v Fourier transform infrared spectrometer equipped with an air-cooled DTGS detector and diamond attenuated total reflection infrared cell at 2 cm^−1^ resolution and 128 scans in the middle−infrared (4000–400 cm^−1^) and far−infrared (600–100 cm^−1^) regions at room temperature. The ATR spectra of 2% solutions of Mn(II) complexes in MeOH, which were obtained by dissolving 1–2 mg complexes in 100 μL MeOH, were measured in the intervals 0, 24, 48, 72 and 96 h. The procedure was conducted by applying a drop on the diamond plate of the ATR accessory. The measurement parameters were the same as before. Instrument control and initial data processing were performed using OPUS Software (v. 7.5, Bruker Optics, Ettlingen, Germany). The FT-Raman spectra of Mn(II) complexes and ligands were collected on a Bruker MultiRam spectrometer (Nd:YAG laser with a CW radiation at 1064 nm) equipped with a liquid N_2_-cooled germanium detector at a resolution of 4 cm^−1^, co-addition of 1024 scans, and laser power values of 300–500 mW.

#### 3.1.3. Thermogravimetry Analysis

Thermogravimetric analysis (TG) was performed on a TGA 4000, PerkinElmer (Waltham, MA, USA). Samples (mass~5.00 mg) were measured in the presence of nitrogen as the furnace atmosphere with a heating rate of 10 °C min^−1^ in the range of 25–1000 °C.

#### 3.1.4. Elemental Analysis

Elemental analysis (C, H and N) of the obtained compounds was carried out on a FLASH 2000 elemental analyzer (Bangkok, Thailand). The program used for data analysis is Thermo Scientic Eager Experience (Waltham, MA, USA), which automatically generates and displays a complete report at the end of each measurement cycle.

#### 3.1.5. UV–VIS Spectroscopy

Electronic absorption spectroscopy was carried out with a UV–Vis spectrophotometer (Agilent Technologies (Santa Clara, CA, USA), Cary300 UVVis).

### 3.2. Biological Activity

#### 3.2.1. Cell Cultures

HT-29 (colorectal adenocarcinoma, morphology: epithelial, ATCC: HTB-38), MCF7 cell line (human breast adenocarcinoma, morphology: epithelial-like, ATCC: HTB-22), A549 cell line (human lung adenocarcinoma, morphology: epithelial, ATCC: CCL-185), PANC-1 (human pancreatic/duct carcinoma, morphology: epithelial, ATCC: CRL-1469) and HaCaT (human keratinocyte, ATCC PCS-200-011) were cultured in Dulbecco’s Modified Eagle’s Medium (DMEM, Corning, NY, USA), supplemented with 10% fetal bovine serum (FBS) and with 1% streptomycin/penicillin. DU145 cell line (human prostate carcinoma) was cultured in minimum essential medium (MEM, Corning) with 10% fetal bovine serum (FBS). Cultures were incubated at 37 °C under a humidified atmosphere containing 5% CO_2_. Cells were passaged using a solution containing 0.05% trypsin and 0.5 mM EDTA. All media and other ingredients were purchased from ALAB, Poland ThermoFisher Scientific (USA).

#### 3.2.2. Cytotoxic Activity

Since most of the studied compounds are insoluble in aqueous media, they needed to be pre-dissolved in DMSO for biological evaluation. DMSO was used in cell cultures at a concentration below 0.1% (*v*/*v*) to minimize its cytotoxicity and changes in gene expression. The cytotoxicity of the studied compounds was assessed using an MTT assay performed according to the protocols described previously [45]. In brief, 1 × 10^4^ cells per well, seeded in a 96-well flat-bottom microtiter plate, were incubated with the tested complexes (**1**, **2**, **3**) at various concentrations for 24 h. After that time, solutions of compounds were washed out; cells were then washed three times with PBS (1X), and a fresh medium was applied. Each compound concentration was tested in five replicates and repeated at least three times. Determined values of IC_50_ (concentration of a drug required to inhibit the growth of 50% of the cells) are given as mean ± S.D.

#### 3.2.3. Cellular Uptake

Metal uptake: Cells HT-29, A549, MCF7, PANC-1 and HaCaT at a density of 2 × 10^6^ cells/2 mL were seeded on 6-well plates and incubated with complexes (c = 1 μM for 24 h) at standard conditions (37 °C, 5% CO_2_). The solutions of the studied complexes **1**, **2** and **3** were removed; the cells were washed twice with PBS (1X), trypsinized, then washed twice with PBS (1X) and then suspended in 1 mL of PBS. The sample was divided into two parts—one for copper uptake investigation and the second one for Bradford Assay. Cells, for ICP-MS analysis, were mineralized in 1 mL of 65% HNO_3_. Measurement of the concentration of ruthenium and iridium ions was determined by a mass spectrometer (ELAN 6100 Perkin Elmer) with an inductively coupled plasma (ICP-MS). Protein content was assessed with Bradford Protein Assay (Thermo Fisher Scientific, Waltham, MA, USA) [65]. The manganese content of the samples was determined by ICP-MS, and the protein content was determined by the Bradford method according to data in the literature [66,67,68,69]. Results were calculated as ng manganese per mg cellular protein from the data obtained in two independent experiments. The experiment was repeated at least three times, and results are presented as mean value ± S.D.

#### 3.2.4. Generation of Reactive Oxygen Species (ROS)

Cellular production of ROS was determined by photometric tests using Cyto-ID^®^Hypoxia/Oxidative Stress Detection Kit (Thermo Fisher) and was carried out as described elsewhere [70]. The assay was performed in 96-well plates, where the PANC-1 cells were seeded at a density of 10^5^ cells per 0.2 mL of medium. The experiments were performed in darkness. All experiments were carried out following the procedures described in our previous papers [70,71].

#### 3.2.5. Detection of Mitochondrial Membrane Potential (ΔΨm)

Mitochondrial membrane potential (ΔΨm) depletion was determined by JC-10 Assay (Life Technologies, Carlsbad, CA, USA) and was carried out as described elsewhere [70]. Briefly, PANC-1 cells were seeded on 96-well plates at 1 × 10^4^ cells per 0.2 mL. After 24 h, the medium was replaced with solutions of inorganic compounds **1**, **2** and **3** at IC_50_ concentrations, as well as gentamicin (0.5 mg mL^−1^) and ciprofloxacin (10 µg mL^−1^) as positive and negative controls, respectively. After that, cells were incubated for 24 h under standard conditions (37 °C, 5% CO_2_). Then, they were washed twice with PBS buffer and incubated with JC-10 for 1 h. Afterward, emissions were measured at two different excitation wavelengths (λ_ex_ = 540 nm, λ_em_ = 570 nm and λ_ex_ = 485 nm, λ_em_ = 530 nm). The results are presented as the intensity ratio of red to green emissions (mean ± S.D.).

#### 3.2.6. Micelles Preparation

Pluronic P-123 micelles loaded with Mn(II) complexes were prepared according to our previously published protocol based on the thin-film hydration method [50]. In brief, 0.25 g of Pluronic P-123 was dissolved in CHCl_3_ at 60 °C, under reflux, for 15 min. In the case of the preparation of loaded micelles, 1 mL of 0.5 mg/mL Mn(II) compounds (**1** or **3**) and 1 mL of CHCl_3_ were added to the hot solution and refluxed for 15 min. Solvent was slowly evaporated (50 °C, 500 mbar, 270 rpm). Then, the thin film was dried under reduced pressure and hydrated under sonication with PBS. The resulting empty or loaded micelles were centrifuged (5000 rpm, 15 min), purified with PBS and lyophilized. The supernatant was used to determine the concentration of non-encapsulated Mn(II) complexes by the ICP-MS technique (Perkin Elmer ELAN 6100). Drug loading content (*LC*) and encapsulation efficiency (*EE*) were calculated using the following equations:

(1) $$LC=\frac{m_{0\;-\;}m_{1}}{m_{t}}\times 100\%\;\;\;EE=\frac{m_{0\;}-\;m_{1}}{m_{0}}\;\times 100\%$$

where *m*_0_—the starting mass of Mn(II) complex before synthesis [g]; *m*_1_—the mass of Mn(II) complex in supernatant after synthesis [g]; and *m_t_*—the total mass of micelles [g].

The size and Zeta potential of the resulting micelles were determined by dynamic light scattering techniques (DLS, ZetaSizer Nano ZS, Malvern Instruments, Malvern, UK). All procedures of the in vitro biological studies involving micelles described in this paper were performed as described above for the Mn(II) complex compounds, unless otherwise indicated.

#### 3.2.7. Interaction with HSA

Human serum albumin (HSA) was dissolved in 10 mM HEPES buffer at pH 7.4. Then the concentration of the solution was determined by UV-Vis spectroscopy. The final concentration of HSA in the sample was 5 × 10^−5^ M. HSA was titrated with compound **1**, **2** or **3** in 20% DMSO (1:1–10 HSA:compound molar ratio). All samples (HSA + compound) were incubated for 1 h at room temperature. The emission spectra for HSA were recorded in the 310–500 nm range (λ_ex_ = 295 nm) using an RF-6000 spectrofluorophotometer (Shimadzu, Tokyo, Japan).

#### 3.2.8. Interaction with Apo-Tf

Apo-transferrin (apo-Tf) was dissolved in 10 mM HEPES buffer at pH 7.4. Then, the concentration of the solution was determined by UV-Vis spectroscopy. The final concentration of apo-Tf in the sample was 3.6 × 10^−6^ M. Apo-Tf was titrated with compound **1**, **2** or **3** in 20% DMSO (1:1–10 apo-Tf:compound molar ratio). All samples (apo-Tf + compound) were incubated for 1 h at room temperature. The emission spectra for apo-Tf were recorded in the 310–500 nm range (λ_ex_ = 295 nm) using an RF-6000 spectrophotometer (Shimadzu).

#### 3.2.9. Interaction with CT-DNA

Calf thymus stock solution (CT-DNA) was prepared by dissolving it in 10 mM HEPES buffer at pH 7.4. Then, the concentration of CT-DNA solution was determined by UV-Vis spectroscopy (ε = 6600 M^−1^cm^−1^ at 258 nm) [72]. The final concentration of CT-DNA in the sample was 5 × 10^−5^ M. CT-DNA solution was mixed with luminescent ethidium bromide in a 1:1 molar ratio and incubated for 1 h at room temperature. EB-CT-DNA complex was incubated for another 1 h with the appropriate compound in 20% DMSO (1:1–10 EB-CT-DNA complex:compound molar ratio). The emission spectra for EB-CT-DNA were recorded in the 520–800 nm range (λ_ex_ = 510 nm) using an RF-6000 spectrophotometer (Shimadzu).

#### 3.2.10. DNA Strand Break Analysis

The ability of compounds **1**, **2** and **3** to induce single- or double-stranded DNA breaks (final DNA concentration per lane was C = 0.31 μg/mL) was tested with the pBR322 plasmid. All compounds (each in 20% DMSO) were dissolved in PBS buffer (pH 7.4). After 1 h of incubation at 37 °C, the reaction mixtures (20 μL) were mixed with 3 μL of loading buffer (bromophenol blue in 30% glycerol) and loaded onto a 1% agarose gel containing ethidium bromide (EB) in TBE buffer (90 mM Tris borate, 20 mM EDTA, pH 7.4). The concentrations given below the electropherogram are the final concentrations. Gel electrophoresis was performed at a constant voltage of 120 V for 120 min. The gel was photographed and developed using a Cleaver Scientific UV transilluminator (Warwickshire, UK).

### 3.3. Molecular Docking

#### 3.3.1. Preparation of the Receptors

**Figure 14:**
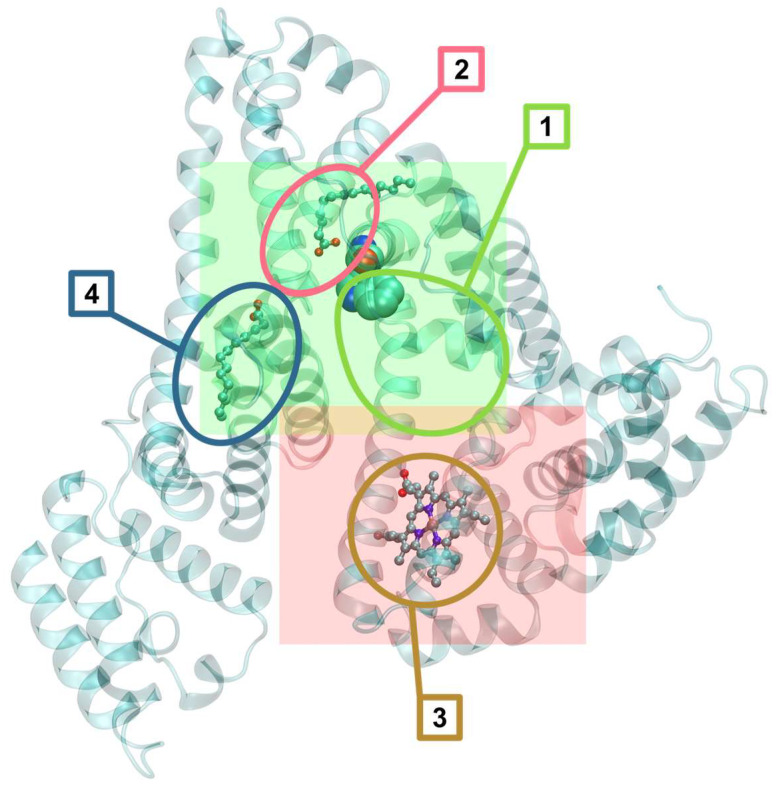
The structure of human serum albumin (PDB entry 1N5U) and the represenative binding sites (outlined and numbered) sampled during docking. Site 1 is near the only Trp 214 residue. Sites 2 and 4 bind lipids in the crystal structure. Site 3 is the heme-binding pocket. Sites 1, 2 and 4 are represented by docking grid HSA1 (shown as the transparent green box), the heme-binding pocket plus some of the HSA surface is encompassed by grid HSA2 (the light red box). The protein structure backbones are shown as cyan-colored transparent secondary structures; the ligands are presented in the crystal structure as ball-and-stick models and the Trp 214 residue of HSA as van der Waals spheres.

The interactions of the studied compounds **1**–**3** with selected biomolecules were evaluated by means of docking with AutoDock4 [73]. The receptor structures, human serum albumin (HSA) available from Protein Data Bank as the entry 1N5U [74], human serum apotransferrin (apo-Tf) from the PDB entry 2HAV [75], and a DNA structure with intercalation binding sites from the entry 1Z3F [76], were prepared as described previously [77], including the recognition of the binding sites. The 1Z3F DNA structure was small enough to be entirely contained within a single docking grid, so the docking sampled the entire molecular surface. HSA and apo-Tf are too big for efficient sampling of the entire molecules, so we selected fragments that were considered most relevant and representative, and only these fragments were sampled. In HSA, there are multiple binding sites, which are presented in Figure 14.

#### 3.3.2. Preparation of the Docked Compounds

Coordinates of the compounds **1**, **2**, and **3** were prepared from their respective crystal structures using default AutoDock Tools (ADT) [73] atom types, apart from the transition metal ions. Parameters for the Mn(II) atom were found on the AutoDock discussion forum [78]. Parameters to calculate Gasteiger atomic partial charges [79] are not available for manganese, and in all cases, ADT produced zero partial charges on metal ions or on the chloride ions bound to Mn^2+^. To assess the proper charge distribution, DFT calculations were performed using ORCA 6.0.1 software [80] for all ligands using the PBE0 density functional [81] and ma-def2-TZVP basis set [82,83]. It was found that the charge distribution significantly depends on the transition metal spin state, and that there is significant charge transfer from the metal ion to its ligand(s), further proving that the Gasteiger charge model is inadequate in this case. The spin multiplicity of Mn^2+^ was selected as a sextet, which was the lowest-energy state. The crystal structures were optimized and the atomic charges calculated based on the PBE0/ma-def2-TZVP level with a Conductor-Like Polarizable Continuum Model [84] implicit solvent model for water; no significant structural changes were observed.

Information on the applicability of charge models other than Gasteiger for docking with AutoDock4 is scarce in the literature, but there is an example of electrostatic potential-derived charges providing a better correlation with experimental results than AutoDock4 models of interactions [85]. Thus, it was decided to calculate the atomic partial charges for all ligands using the well-established RESP method [86]. The fitting of the RESP charges was performed with MultiWFN software (http://sobereva.com/multiwfn/index.html, access: 12 March 2026) [87,88]. AutoDock4 represents docked molecules as united atomic models without nonpolar hydrogen atoms. The RESP charges were therefore fitted to the molecular electrostatic potential, with the constraints enforcing zero charges on such hydrogen atoms; also, the charges on atoms equivalent to the identical ligand molecules coordinated to the manganese ion were constrained to be equal. A comparison of the performance of the Gasteiger and RESP charges was performed for docking of **3** against the 1Z3F DNA structure. It was found that the incorrect neutral charges assigned to manganese and chloride anions with ADT (due to the lack of Gasteiger parameters) caused significant artifacts, suggesting that **3** can both intercalate and bind to the minor groove. With the proper negative charges on the chloride anions, as calculated with the RESP method, the minor groove binding mode is impossible due to repulsive interactions with the phosphate backbone, and only partial intercalation of one of the aromatic rings of **3** can be observed.

#### 3.3.3. Docking Setup

All the docking grids used the default AutoGrid4 [73] parameters: point spacing 0.375 Å, 0.5 Å smoothing distance, and distance-dependent dielectric constant factor −0.1465. The DNA docking grid was centered on the DNA center of mass and sized 30 × 30 × 37 Å, with the largest size along the double helix axis.

The HSA receptor was represented by 2 docking grids, shown in Figure 14. The potential map grid HSA1 map was centered on the Trp 214 residue and sized 33.75 × 30 × 30 Å relative to the 1N5U crystal coordinates. It covered a large binding pocket next to the Trp 214 residue (site 1 in Figure 14) and 2 lipid binding sites (numbered 2 and 4 in Figure 14). The second grid HSA2, centered on the heme iron and sized 33.75 × 36.25 × 36.25 Å encompassed the heme-binding pocket (site 3 in Figure 14) and some of the HSA surface clefts.

**Figure 15:**
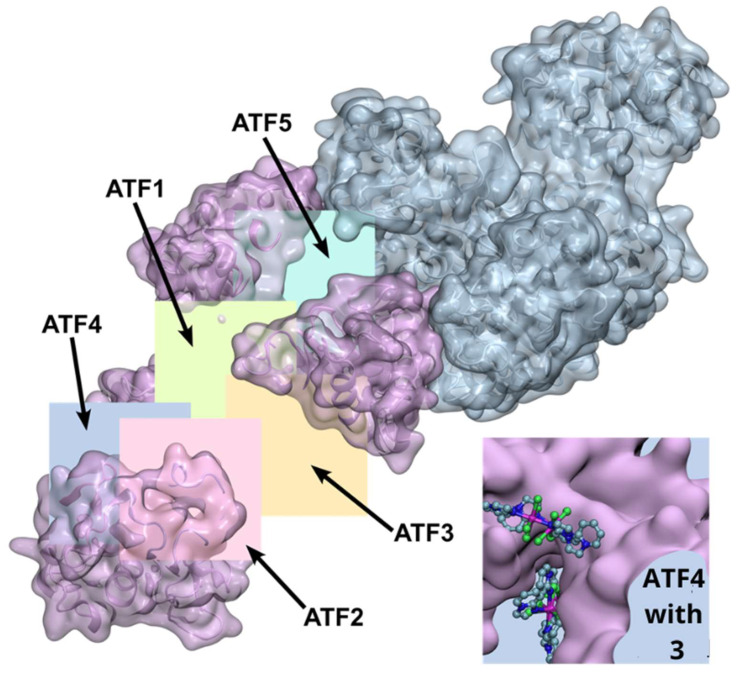
The crystal structure of apotransferrin apo-Tf (PDB entry 2HAV) showing the extents of the docking grids encompassing the identified binding sites. Binding site 5, represented by the grid ATF5, is between chain A (magenta) and B (cyan). The inset shows the protein fragment represented by ATF4 with representative binding poses of **3**.

To adequately represent the multiple possible binding pockets [77] of the large apo-Tf structure (Figure 15), as many as 5 cubic grids were used, centered at −36.325, −1.776, and 0.665 (grid ATF1), at −42.818, −7.435, and −21.026 (grid ATF2), at −23.178, −4.200, and −12.921 (grid ATF3), at −55.864, −3.726, and −18.111 (grid ATF4), and at −21.809, 4.632, and 17.431 (grid ATF5). All these grids had the edge length of 26.25 Å and the default spacing as described above. It should be noted that the grid ATF4 does not represent a single defined binding site but instead a deep and long cleft on the apo-Tf surface. It is shown as the inset in Figure 15.

The docking used the Lamarckian Genetic Algorithm with default parameters [73] unless stated otherwise. For every grid and ligand, 300 independent docking runs were performed, and the representative clusters were identified by means of the coordinate RMSD difference threshold of 2 Å.

## 4. Conclusions

In this study, we investigated the structural and biological properties of new manganese(II) complexes with imidazo[1,2-a]pyridine and its chloro- and bromo- derivatives. Two of them, **1** and **2**, crystallize in the triclinic space group P$\bar{1},\;$while **3** crystallizes in the monoclinic space group P2_1_/c, respectively. Complex **3**, a chloro-bridged manganese(II) coordination polymer, described by us, has a relatively rare motif. Thermal analysis showed that the gradual mass loss observed in the temperature range 20–1000 °C ultimately leads to the formation of MnO_2_ as the final decomposition product.

The stability of complexes **1**–**3** in solution was investigated using UV-Vis and FT-IR spectroscopy. Overall, the combined spectroscopic and computational results suggest that complexes **1** and **2** may undergo partial structural rearrangement in solution. The spectral similarities observed for these systems indicate that the formation of μ-Cl-bridged manganese(II) species cannot be excluded. In particular, the solution structures of complexes **1** and **2** may resemble chloro-bridged coordination motifs analogous to those identified in complex **3**, suggesting the possible formation of polymeric manganese(II) assemblies that remain reasonably stable under the studied conditions.

Guided by these observations, the [Mn(3-Ximpy)_2_Cl_4_]^2−^ model was employed in the computational analysis, as it captures the key features of the μ-Cl-bridged coordination environment implied by the spectroscopic data. Importantly, the calculations were intended to represent the likely solution behavior of complexes **1** and **2** rather than to demonstrate the existence of a fully discrete [Mn(3-Ximpy)_2_Cl_4_]^2−^ species.

Interestingly, despite their similar coordination cores and spectroscopically supported coordinated forms in solution, the complexes exhibit markedly different biological activities. The most active complex, **2**, contains a bromine substituent in the imidazo[1,2-a]pyridine ring, which enhances lipophilicity, membrane permeability, and cellular uptake, leading to higher accumulation in PANC-1 cells, increased ROS generation, and mitochondrial dysfunction. In contrast, complexes **1** and **3** exhibit lower uptake and weaker cytotoxic effects. These findings indicate that substituent effects, hydrophobicity, and interactions with biomolecules, such as transport-related proteins, play a crucial role in determining biological activity, beyond coordination geometry alone.

Molecular docking studies, performed using the spectroscopically supported coordinated forms, showed moderate interactions with serum proteins (HSA and apo-Tf), with preferred binding sites located at protein interfaces. In contrast, interactions with DNA were weak and limited to partial intercalation of a single aromatic ring, while complex **3** even showed unfavorable interactions. These results are consistent with experimental observations and indicate that DNA is unlikely to be the primary biological target.

Encapsulation of complexes **1** and **3** into Pluronic P-123 micelles significantly enhanced cellular uptake and cytotoxicity while maintaining selectivity. Overall, Mn(II) complexes, particularly complex **2**, represent promising candidates for further development as protein-associated, ROS-mediated anticancer agents.

## Figures and Tables

**Table 1:** Values of IC_50_ [µM] (concentration of a drug required to inhibit the growth of 50% of the cells) for HT-29, A549, MCF7, DU145, PANC-1, HaCaT cells after 24 h with the studied compounds and cisplatin (CDDP) as reference.

	IC50 [μM]					
Compound	HT-29	MCF-7	Du-145	A549	PANC-1	HaCat
**1**	>100	>100	>100	>100	51.90 ± 6.2	>100
**2**	>100	>100	>100	>100	27.82 ± 3.7	>100
**3**	>100	>100	>100	>100	51.47 ± 4.1	>100
**imidazo[1,2-a]pyridine**	>100	>100	>100	>100	>100	>100
**3-chloroimidazo[1,2-a]pyridine**	>100	>100	>100	>100	>100	>100
**3-bromo[imidazo[1,2-a]pyridine**	>100	>100	>100	>100	>100	>100
CDDP	132.9 ± 6.21	56.9 ± 3.51	65.3 ± 5.65	59.0 ± 4.36	98.6 ± 4.68	46.3 ± 4.13

**Table 2:** Loading content, encapsulation efficiency, hydrodynamic diameter and Zeta potential for the investigated Pluronic P-123 formulations.

Formulation	Loading Content ± SD [%]	Encapsulation Efficiency ± SD [%]	Hydrodynamic Diameter ± SD [nm]	Zeta Potential ± SD [mV]
**1_M**	21.65 ± 3.27	86.52 ± 1.23	29 ± 7 (PDI = 0.4)	−1.59 ± 0.33 mV (pH = 7.4)
**3_M**	36.01 ± 8.43	92.20 ± 0.39	31 ± 6 (PDI = 0.3)	−1.59 ± 0.33 mV (pH = 7.4)

**Table 3:** IC_50_ values for PANC-1 and HaCaT cell lines determined after 24 h incubation with **1_M** and **3_M** and additional 24 h for recovery time in free media.

Formulation	IC_50_ ± SD	
	PANC-1	HaCaT
**1_M**	24.57 ± 11.20 μM (0.24 ± 0.11 mg/mL micelles)	470.69 ± 140.19 μM (4.53 ± 1.35 mg/mL micelles)
**3_M**	13.15 ± 4.41 μM (0.03 ± 0.01 mg/mL micelles)	349.59 ± 88.21 μM (0.75 ± 0.19 mg/mL micelles)

**Table 4:** The AutoDock4 binding scores [kcal/mole] of the studied manganese complexes to the DNA B double helix, for all clusters of the docked poses.

Complex	1	2	3
**# of clusters:**	2	2	3
**Best:**	−0.62	−0.73	+0.33
**Worst:**	−0.59	−0.71	+0.42

## Data Availability

The data supporting this article have been included as part of the Supplementary Information.

## Supplementary Materials

The following supporting information can be downloaded at https://www.mdpi.com/article/10.3390/molecules31061007/s1. It includes additional experimental details: X-ray crystallographic data with selected bond lengths and angles, short intra- and intermolecular contacts, and π–π stacking interactions; FT-IR and Raman spectra; UV-Vis and FT-IR spectra in solution (MeOH/DMEM); thermoanalytical data; and molecular docking results, including representative binding poses of Mn(II) complexes **1**–**3** with human serum albumin and apo-transferrin (DOC) [12,13,14,15,16,17,18,19,20,21,22,23,24,25,26,27].
