# Supplementary material for: Substituent Effects Control the Biological Activity of Mn(II) Imidazo[1,2-a]pyridine Complexes
Source: Molecules. 2026 Mar 17;31(6):1007. doi: 10.3390/molecules31061007 (PMC13028769; doi:10.3390/molecules31061007)
Supplement: Supplementary file 1 [file molecules-31-01007-s001.zip › molecules-4182854-supplementary.pdf]

Electronic Supplementary Information (ESI)

Substituent Effects Control the Biological Activity of Mn(II) Imidazo[1,2-a]pyridine Complexes

Magdalena Rydz [1,*], Tomasz Mazur [1], Anna Świtlicka [2], Urszula K. Komarnicka [3], Daria Wojtala [3], Monika K. Lesiów [3], Agnieszka Kyzioł [4], Paweł Kędzierski [1] and Dariusz C. Bieńko [1]

[1] Faculty of Chemistry, Wroclaw University of Science and Technology, Wybrzeze Wyspianskiego 27, 50-370 Wroclaw, Poland
[2] Department of Crystallography, Institute of Chemistry, University of Silesia, Szkolna 9, 40-006 Katowice, Poland
[3] Faculty of Chemistry, University of Wroclaw, F. Joliot-Curie 14, 50-383 Wroclaw, Poland
[4] Faculty of Chemistry, Jagiellonian University, Gronostajowa 2, 30-387 Krakow, Poland
* Correspondence: magdalena.rydz@pwr.edu.pl; Tel.: +48-71-320-24-48

Contents:

Figures:

Figure S1. Thermogravimetric, TG (black) and differential thermal analysis(DTG, red) curves of complexes **1-3.**

Figure S2. The comparison of the FT-IR spectra of **1-3** in the 3600-450 cm^{-1} spectral range.

Figure S3. The comparison of the Raman spectra of **1-3** in the 3200-100 cm^{-1} spectral range.

Figure S4. The comparison of the FT-IR and Raman spectra of **1-3** in the 500-50 cm^{-1} spectral range.

Figure S5a. UV-VIS spectra of complexes **1-3** in DMEM after 0h, 24h, 48h and 72h.

Figure S5b. UV-VIS spectra of complexes **1-3** in MeOH after 0h, 24h, 48h and 72h.

Figure S6. FT-IR spectra of complexes **1-3** in solid state and after dissolved (*) in MeOH for 0h, and 96h.

Figure S7. Representative binding poses of **1** to the sites of human serum albumin. The protein structure presented as transparent cyan backbone, the ligand poses as ball and stick models. Elements color coded: C-cyan, N-deep blue, O-red, P-golden, Cl-green, Mn-purple.

Figure S8. Representative binding poses of **3** to the sites of human serum albumin. The protein structure presented as transparent cyan backbone, the ligand poses as ball and stick models, with atom colors as in Figure S7.

Figure S9. Representative binding poses of **1** at the sites of human serum apo-transferrin. The protein structure is shown as a transparent gray surface with a colored backbone: chain A is in magenta, and chain B is in cyan. The ligand poses are depicted as ball-and-stick models, with atom colors as shown in Figure S7.

Figure S10. Representative binding poses of **2** at the sites of human serum apo-transferrin. The protein structure is shown as a transparent gray surface with a colored backbone: chain A is in magenta, and chain B is in cyan. The ligand poses are depicted as ball-and-stick models, with atom colors as shown in Figure S7.

Figure S11. Agarose gel electrophoresis of pBR322 plasmid cleavage by compounds 1, 2 and 3 (each in 20% DMSO).

Tables:

Table S1. Crystal data and structure refinement for **1-3.**

Table S2. Selected bond lengths (Å) and angles (deg) for **1-3**.

Table S3. Doubled chloro-bridged manganese(II) coordination polymers (Cambridge Structure Database).

Table S4. Short intra– and intermolecular contacts in **1–3**.

Table S5. Short π•••π stacking interactions in **1–3**.

Table S6. Thermoanalytical data for complexes **1-3**.

Table S7. The AutoDock4 scores of binding [kcal/mole] of the studied manganese complexes (**1-3**) to selected binding sites of the human serum albumin. Site numbering as in Figure 7 (see paper). The scores within kT from the best result shown on gray background.

Table S8. The AutoDock4 scores of binding [kcal/mole] of the studied manganese complexes **1-3** to selected binding sites of the apo structure of the human serum apotransferrin, apo-Tf. Site numbering as in Figure 1 (see paper). The scores within kT from the best result shown on gray background.

Complex 1

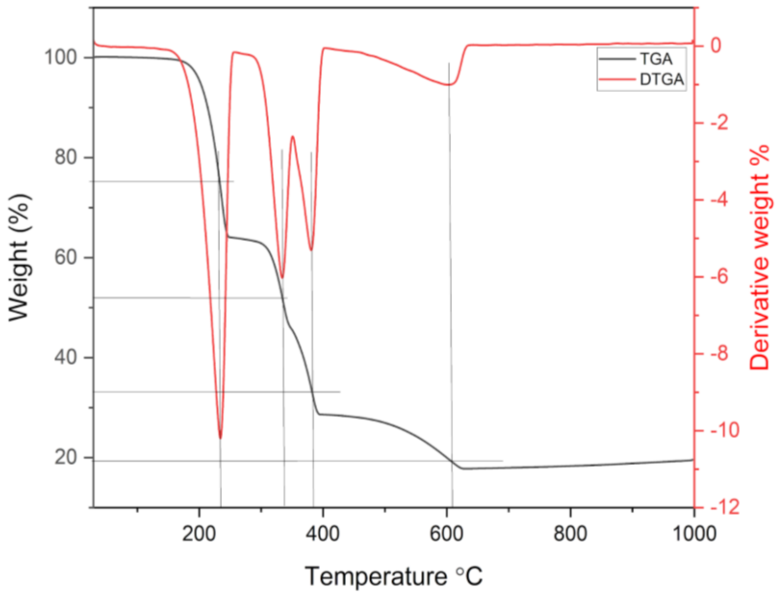

Complex 2

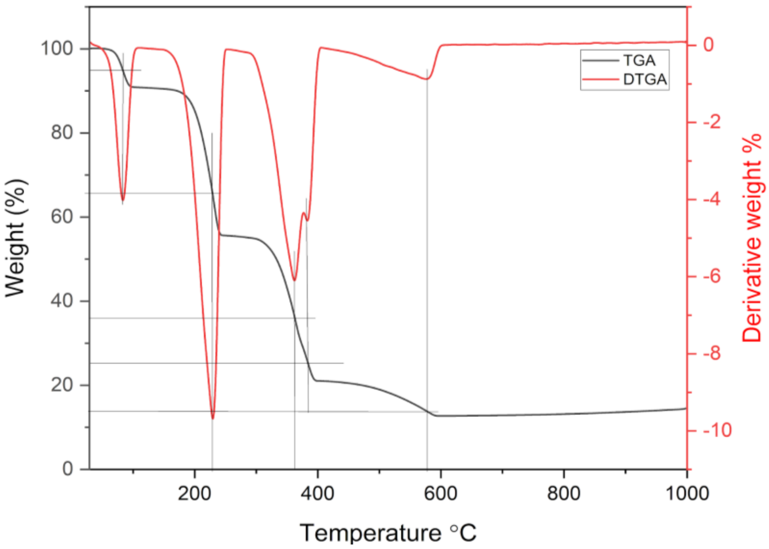

Complex 3

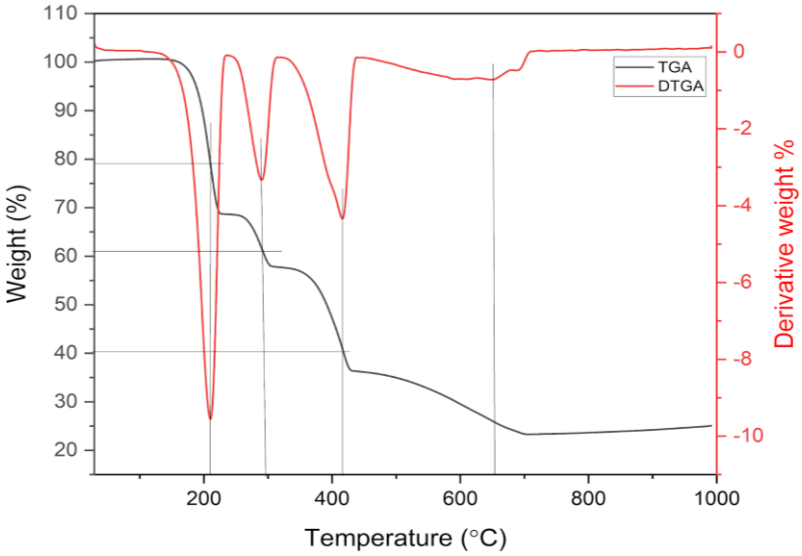

Figure S1. Thermogravimetric, TG (black) and differential thermal analysis(DTG, red) curves of complexes **1-3.**

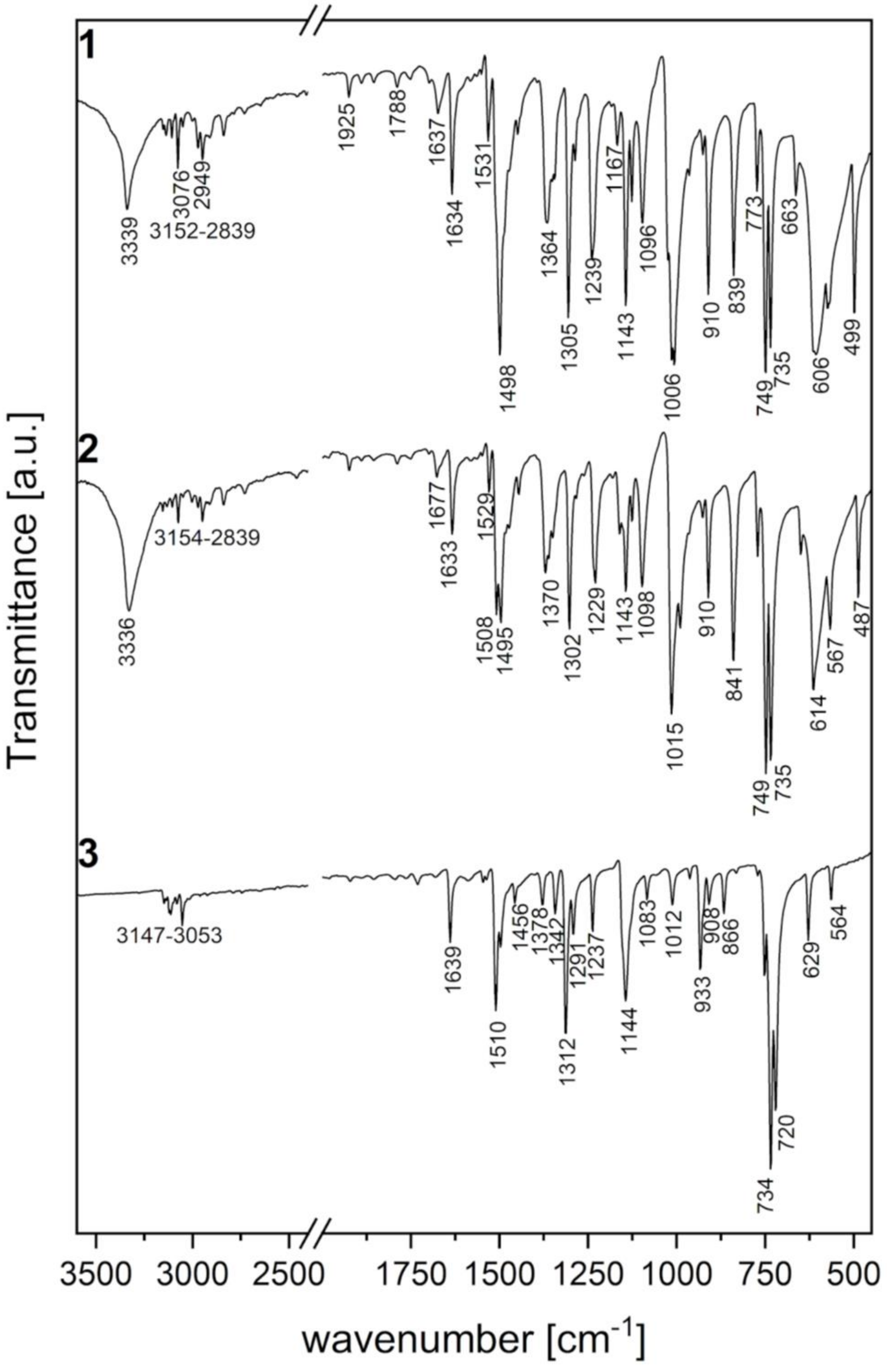

Figure S2. The comparison of the FT-IR spectra of **1-3** in the 3600-450 cm^{-1} spectral range.

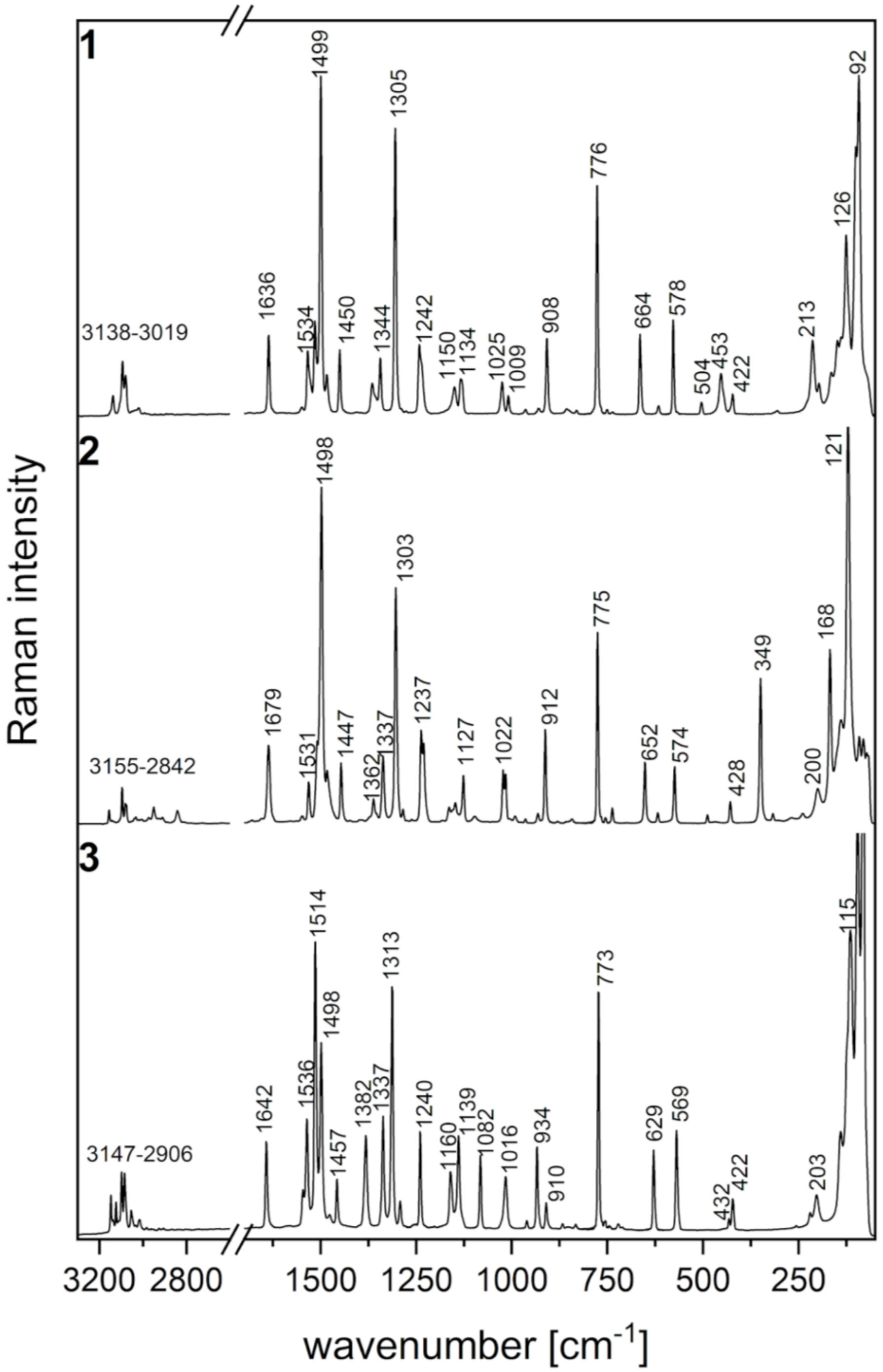

Figure S3. The comparison of the Raman spectra of **1-3** in the 3200-100 cm^{-1} spectral range.

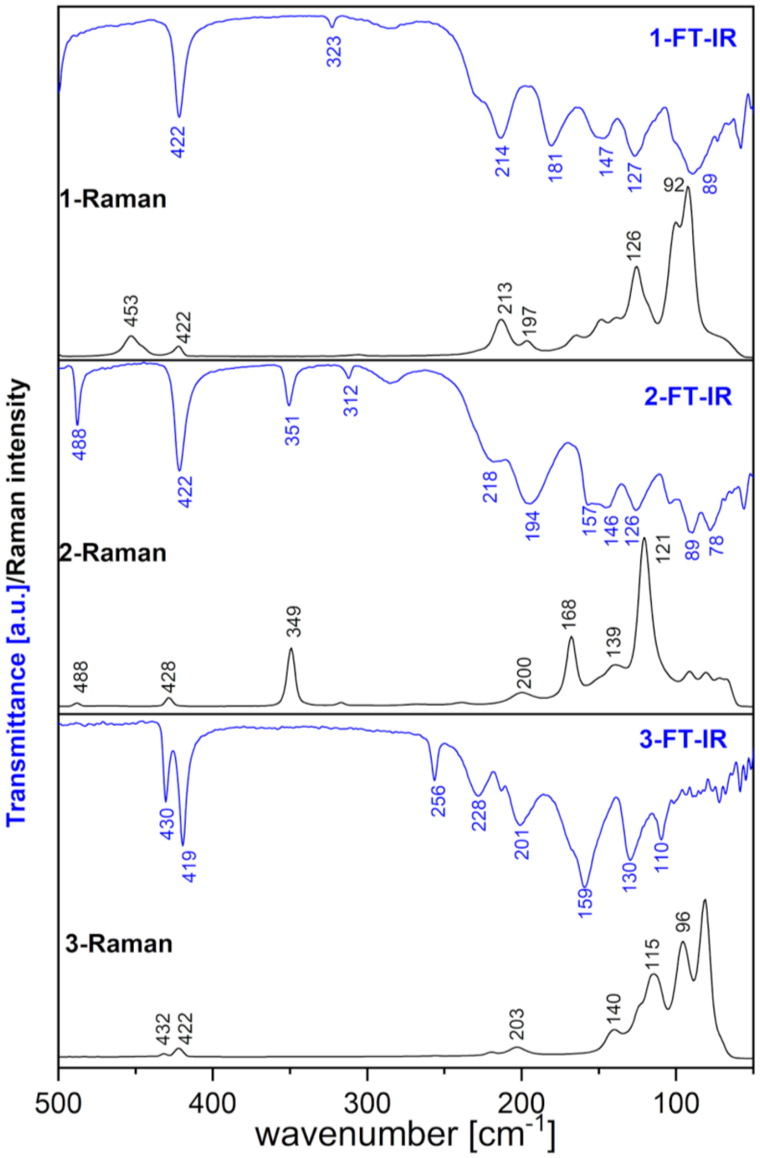

Figure S4. The comparison of the FT-IR and Raman spectra of **1-3** in the 500-50 cm^{-1} spectral range.

Complex 1

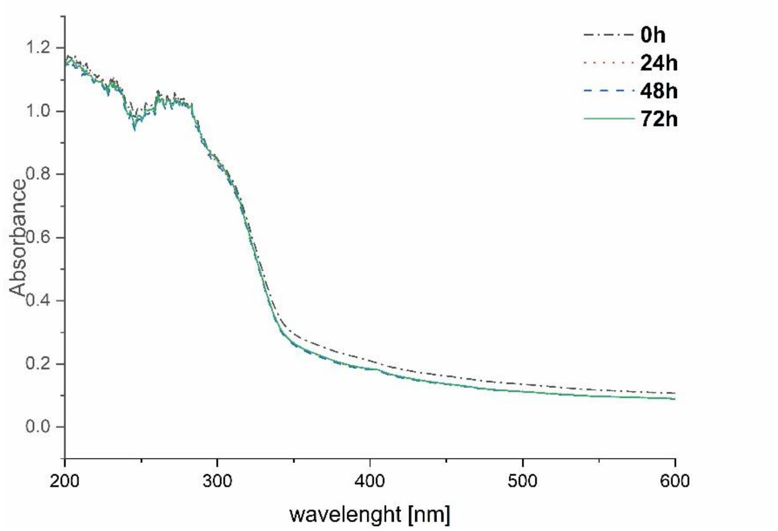

Complex 2

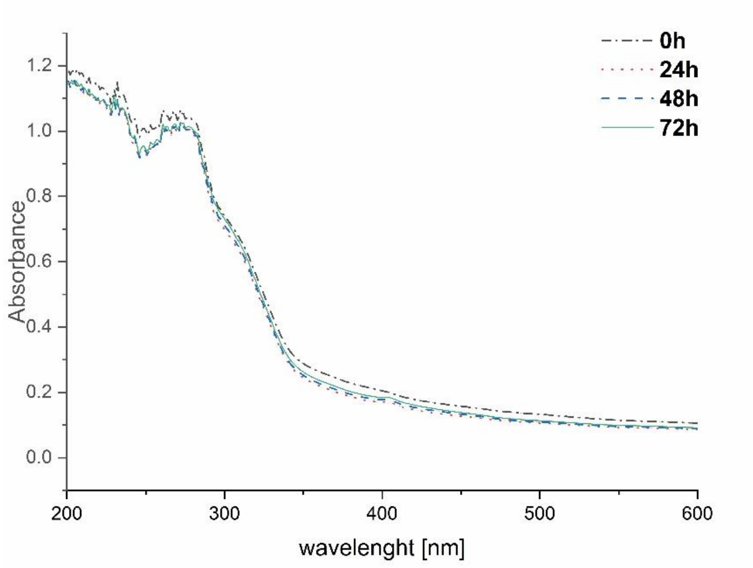

Complex 3

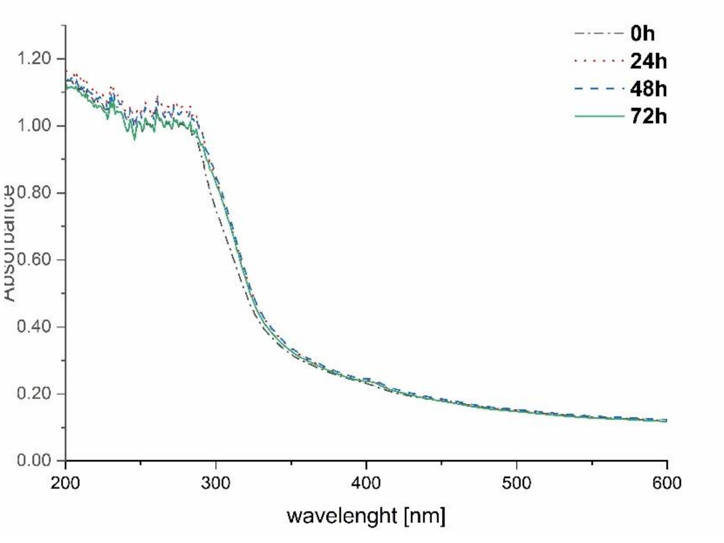

Figure S5a. UV-VIS spectra of complexes **1-3** in DMEM after 0h, 24h, 48h and 72h.

Complex 1

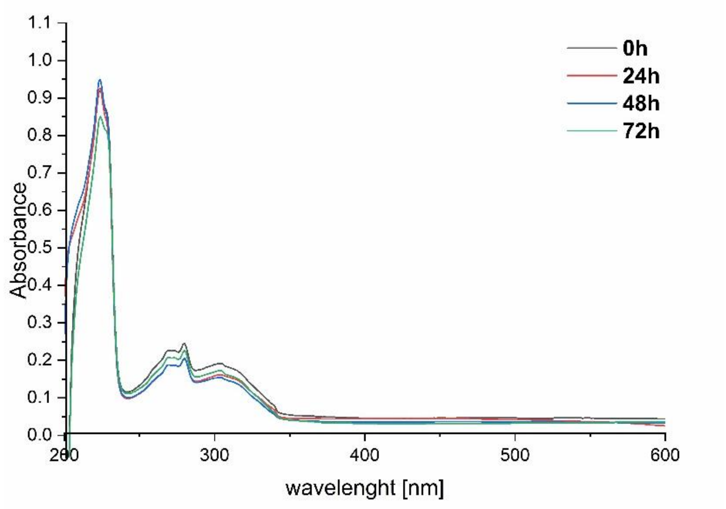

Complex 2

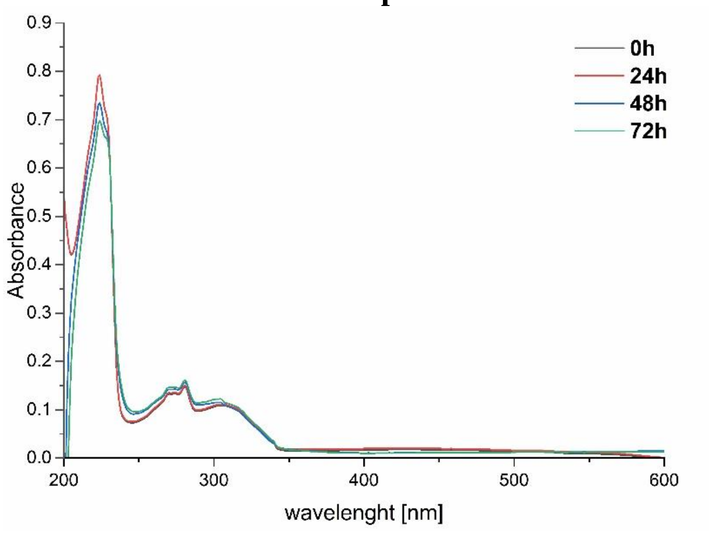

Complex 3

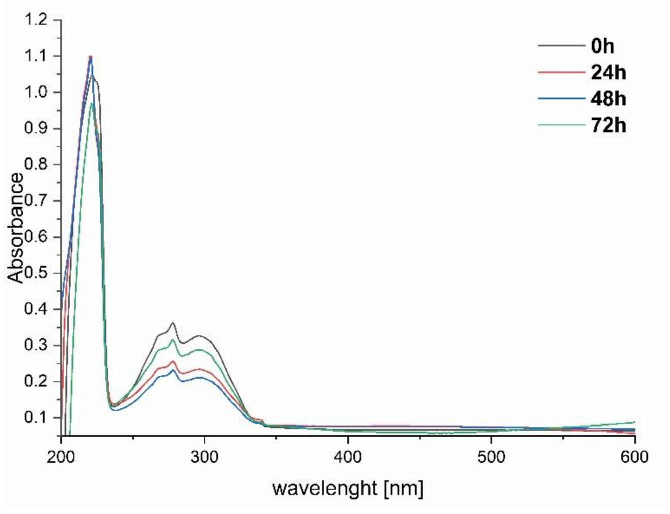

Figure S5b. UV-VIS spectra of complexes **1-3** in MeOH after 0h, 24h, 48h and 72h.

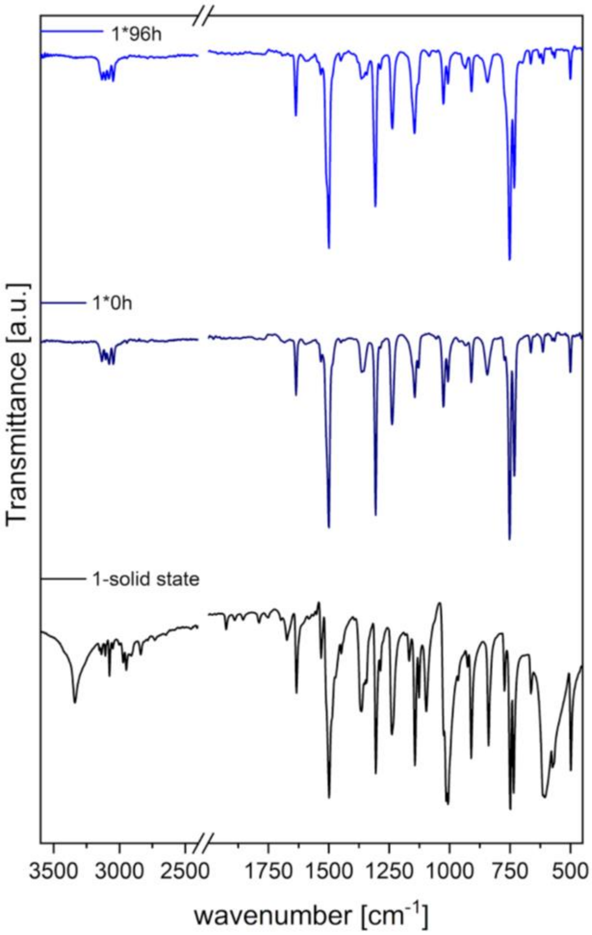
1*96h
1*0h
1-solid state
Transmittance [a.u.]
3500 3000 2500 1750 1500 1250 1000 750 500
wavenumber [cm-1]

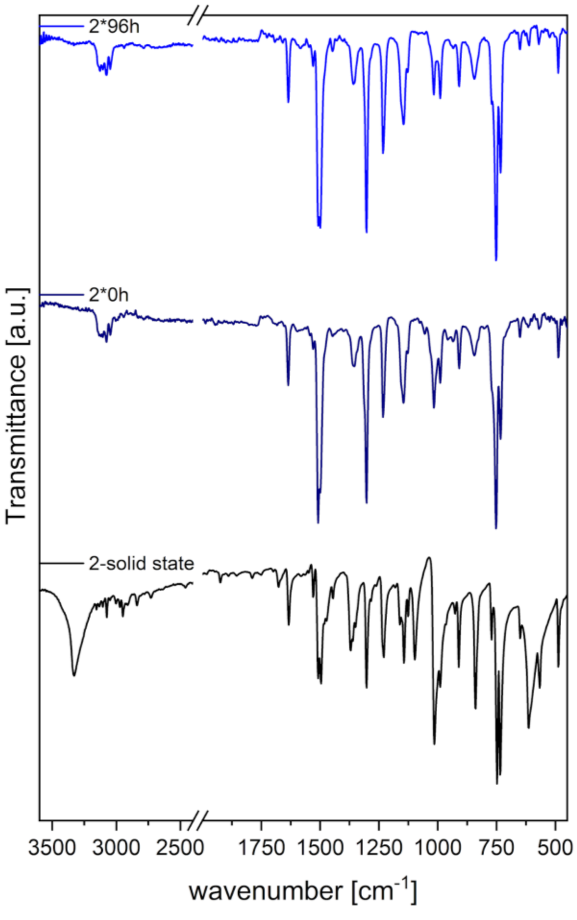
2*96h
2*0h
2-solid state
Transmittance [a.u.]
3500 3000 2500 1750 1500 1250 1000 750 500
wavenumber [cm-1]

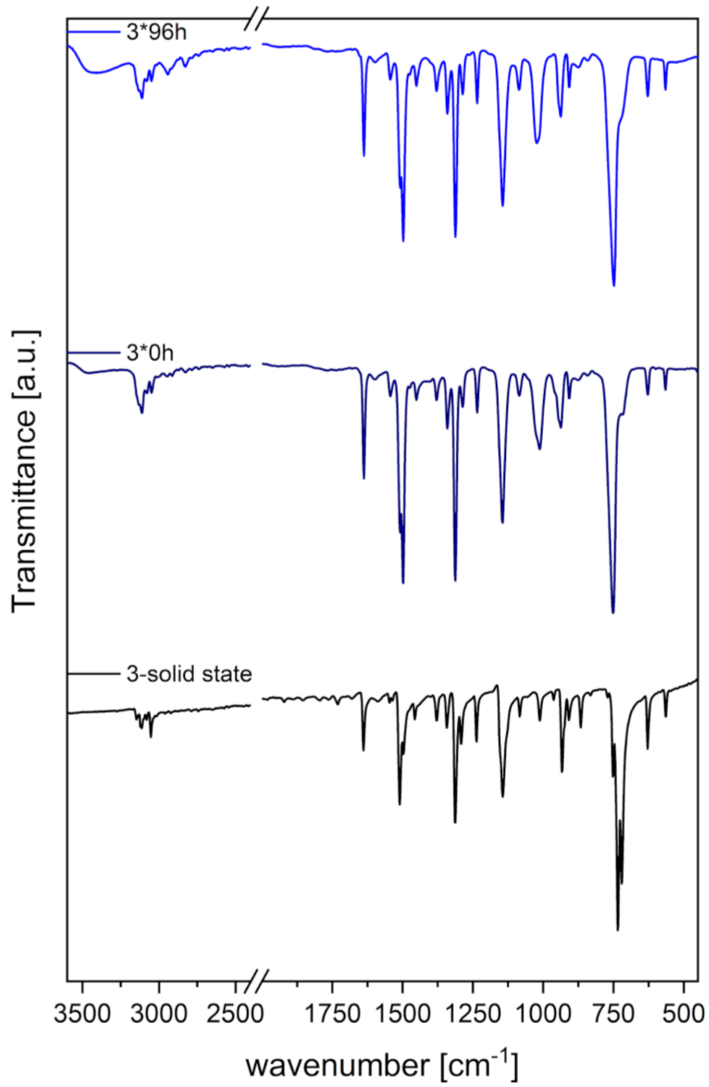

Figure S6. FT-IR spectra of complexes **1-3** in solid state and after dissolved (*) in MeOH for 0h, and 96h.

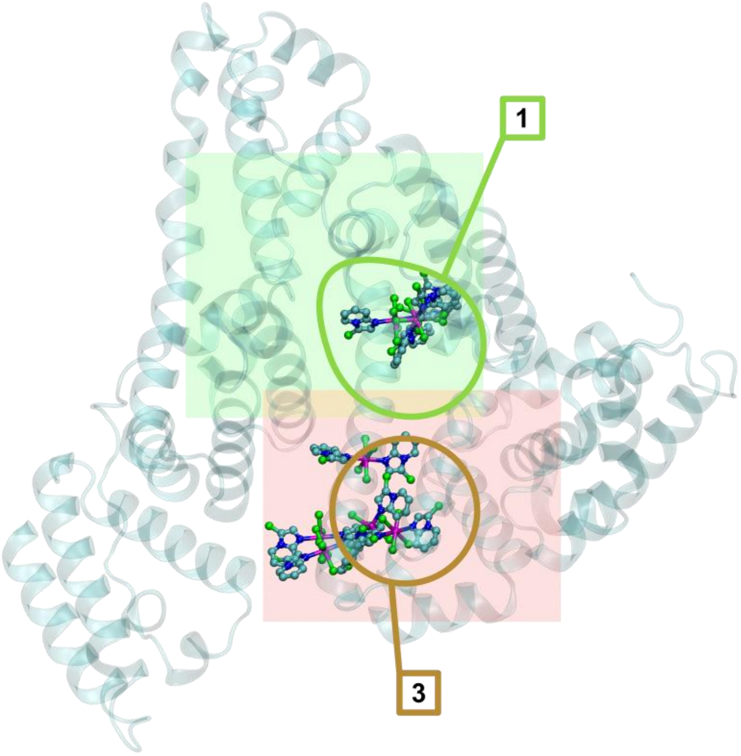

Figure S7. Representative binding poses of **1** at the binding sites of human serum albumin. The protein structure is shown as a transparent cyan backbone, and the ligand poses are displayed as ball-and-stick models. Elements are color-coded as follows: C – cyan, N – deep blue, O – red, P – gold, Cl – green, Mn – purple.

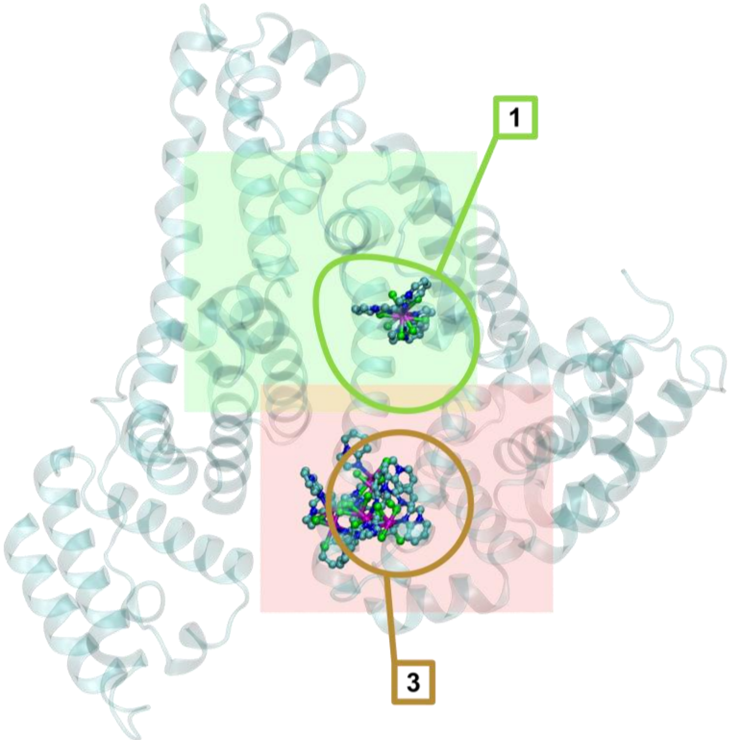

Figure S8. Representative binding poses of **3** to the sites of human serum albumin. The protein structure presented as transparent cyan backbone, the ligand poses as ball and stick models, with atom colors as in Figure S7.

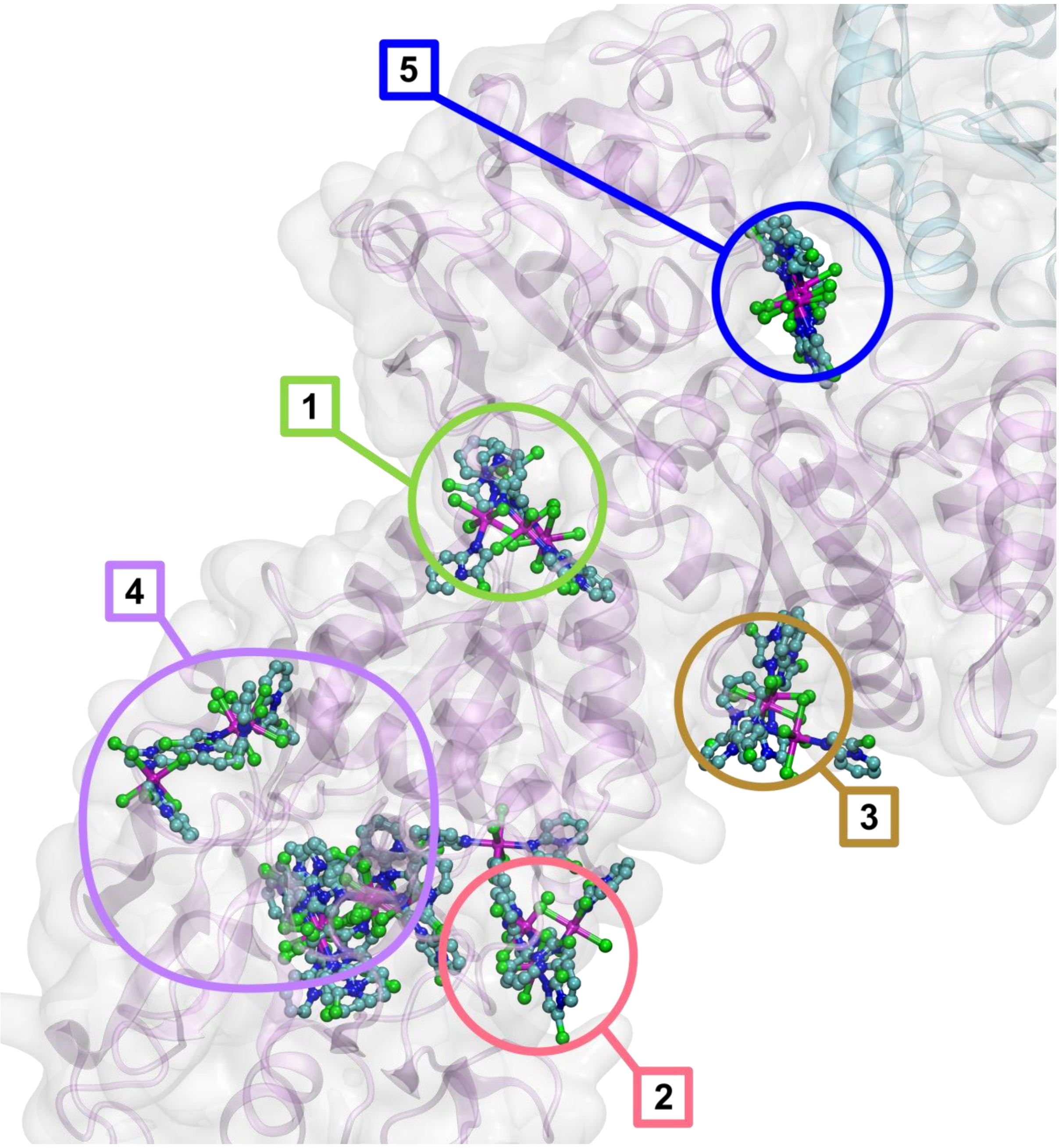

Figure S9. Representative binding poses of **1** at the sites of human serum apo-transferrin. The protein structure is shown as a transparent gray surface with a colored backbone: chain A is in magenta, and chain B is in cyan. The ligand poses are depicted as ball-and-stick models, with atom colors as shown in Figure S7.

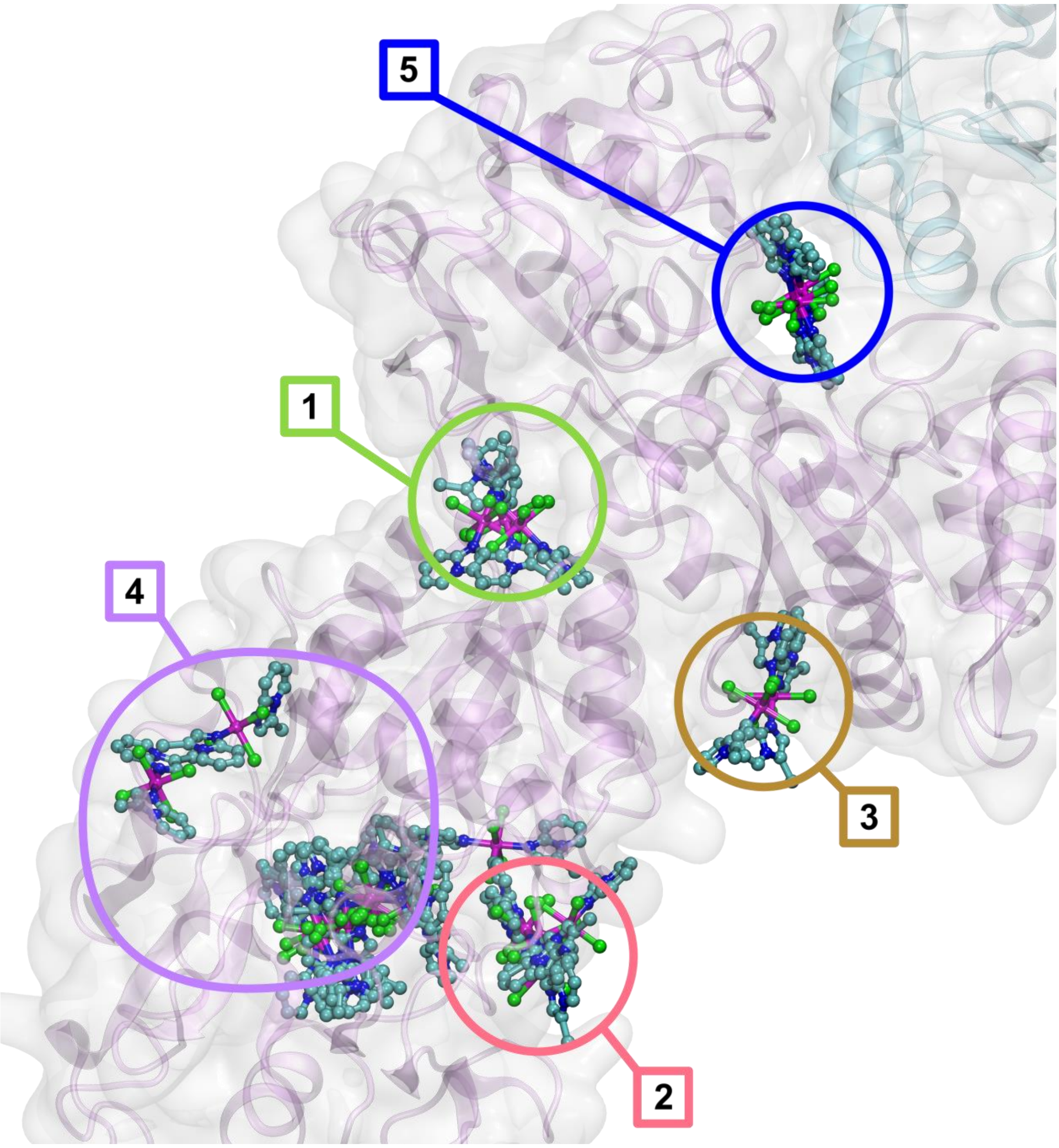

Figure S10. Representative binding poses of **2** at the sites of human serum apo-transferrin. The protein structure is shown as a transparent gray surface with a colored backbone: chain A is in magenta, and chain B is in cyan. The ligand poses are depicted as ball-and-stick models, with atom colors as shown in Figure S7.

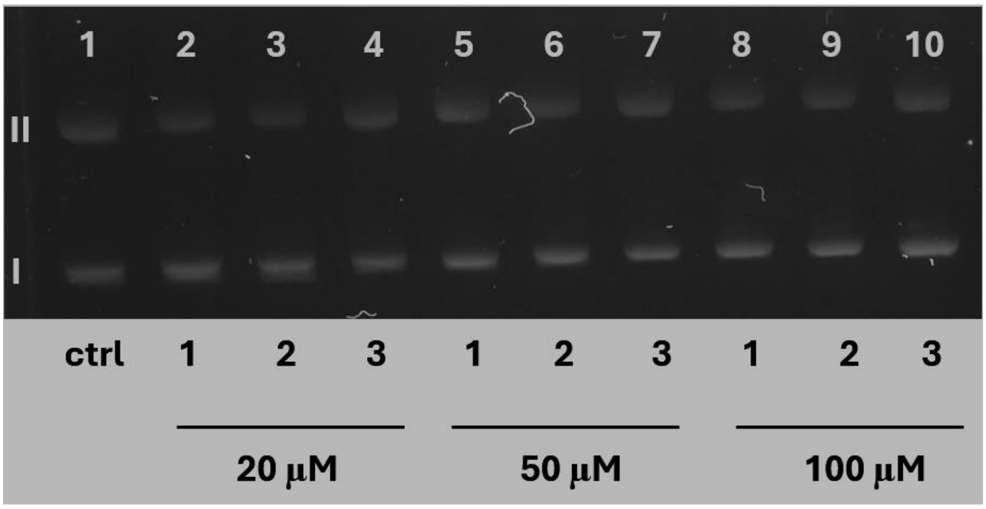

Figure S11. Agarose gel electrophoresis of pBR322 plasmid cleavage by compounds 1, 2 and 3 (each in 20% DMSO).

Table S1. Crystal data and structure refinement for **1-3.**

	**1**	**2**	**3**
Empirical formula	$C_{16}H_{18}Cl_4O_2N_4Mn$	$C_{16}H_{18}N_4O_2Cl_2Br_2Mn$	$C_{14}H_{12}Cl_2N_4Mn$
Formula weight	495.08	584.00	362.12
Temperature [K]	293.0(2)	293.0(2)	293.0(2)
Wavelength [Å]	0.71073	0.71073	0.71073
Crystal system	triclinic	triclinic	monoclinic
Space group	$P\underline{1}$	$P\underline{1}$	$P2_1/c$
Unit cell dimensions [Å, °]	a = 6.6231(6)	a = 6.6335(8)	a = 3.7524(4)
	b = 8.4448(8)	b = 8.6417(8)	b = 14.3668(13)
	c = 9.6677(8)	c = 9.6936(11)	c = 13.3913(9)
	α = 71.283(8)	α = 70.355(9)	
	β = 72.474(7)	β = 72.882(10)	β = 93.705(7)
	γ = 87.328(7)	γ = 86.270(9)	
Volume [Å ^{3}]	487.60(8)	499.80(10)	720.42(11)
Z	1	1	2
Density (calculated) [Mg/m^3]	1.686	1.940	1.669
Absorption coefficient [mm^{-1}]	1.245	4.945	1.283
F(000)	251	287	366
Crystal size [mm]	0.28 x 0.23 x 0.16	0.27 x 0.18 x 0.13	0.13 x 0.06 x 0.05
θ range for data collection [°]	3.86 to 25.05	3.96 to 25.05	3. to 25.05
Index ranges	-9 δ *h* δ 8	-6 δ *h* δ 9	-4 δ *h* δ 4
	-10 δ *k* δ 11	-11 δ *k* δ 11	-19 δ *k* δ 13
	-11δ *l* δ 13	-13 δ *l* δ 13	-18 δ *l* δ 13
Reflections collected	3919	4382	3793
Independent reflections	2315 (R_{int} = 0.0307)	2351 (R_{int} = 0.0740)	1707 (R_{int} = 0.0788)
Completeness to 2θ [%]	99.7	99.7	99.5
Data / restraints / parameters	2315/ 0 / 127	2351/ 3 / 129	1707 / 0 /97
Goodness-of-fit on F^2	1.041	1.033	1.175
Final R indices [*I* > 2 ʃ(*I*)]	R_1 = 0.0316	R_1 = 0.0637	R_1 = 0.0777
	wR_2 = 0.0744	wR_2 = 0.1635	wR_2 =0.2031
R indices (all data)	R_1 = 0.0431	R_1 = 0.0796	R_1 = 0.0946
	wR_2 = 0.0811	wR_2 = 0.1807	wR_2 =0.2150
Largest diff. peak and hole [eÅ^{-3}]	0.298 and -0.328	-1.467 and 1.263	-0.510 and 1.882

Table S2. Selected bond lengths (Å) and angles (deg) for **1**–**3.**

**1**			
Bond lengths [Å]		Bond angles [°]	
Mn(1)–N(1)	2.2494(15)	N(1)–Mn(1)–N(1)a	180.0
Mn(1)–N(1)a	2.2494(15)	N(1)–Mn(1)–N(1)	88.61(6)
Mn(1)–O(1)	2.2450(14)	N(1)–Mn(1)–O(1)a	91.39(6)
Mn(1)–O(1)a	2.2450(14)	N(1)a–Mn(1)–O(1)	91.39(6)
Mn(1)–Cl(1)	2.5204(5)	N(1a)– Mn(1)–O(1)a	88.61(6)
Mn(1)–Cl(1)a	2.5204(5)	N(1)–Mn(1)–Cl(1)	88.64(4)
		N(1)–Mn(1)–Cl(1)a	91.36(4)
		N(1)a–Mn(1)–Cl(1)	91.36(4)
		N(1)a–Mn(1)–Cl(1)a	88.64(4)
		O(1)–Mn(1)–Cl(1)	93.57(3)
		O(1)a–Mn(1)–Cl(1)	86.43(3)
		O(1)a–Mn(1)–Cl(1)a	93.57(3)
		O(1)–Mn(1)–Cl(1)a	86.43(3)
		O(1)a–Mn(1)–O(1)	180.0
		Cl(1)–Mn(1)–Cl(1)	180.0

**2**			
Mn(1)–N(1)<br>Mn(1)–N(1)b<br>Mn(1)–O(1)<br>Mn(1)–O(1)b<br>Mn(1)–Cl(1)<br>Mn(1)–Cl(1)b	2.254(4)<br>2.254(4)<br>2.250(4)<br>2.250(4)<br>2.5388(13)<br>2.5388(13)	N(1)–Mn(1)–N(1)b<br>N(1)–Mn(1)–O(1)<br>N(1)–Mn(1)–O(1)b<br>N(1)b–Mn(1)–O(1)<br>N(1)b– Mn(1)–O(1)b<br>N(1)–Mn(1)–Cl(1)<br>N(1)–Mn(1)–Cl(1)b<br>N(1)b–Mn(1)–Cl(1)<br>N(1)b–Mn(1)–Cl(1)b<br>O(1)–Mn(1)–Cl(1)<br>O(1)b–Mn(1)–Cl(1)<br>O(1)b–Mn(1)–Cl(1)b<br>O(1)–Mn(1)–Cl(1)b<br>O(1)b–Mn(1)–O(1)<br>Cl(1)–Mn(1)–Cl(1)	180.0(2)<br>88.55(16)<br>91.45(16)<br>91.45(16)<br>88.54(16)<br>89.41(12)<br>90.59(12)<br>90.59(12)<br>89.41(12)<br>93.25(10)<br>86.75(10)<br>93.25(10)<br>86.75(10)<br>180.0<br>180.00(6)
**3**			
Mn(1)–N(1)<br>Mn(1)–N(1)a<br>Mn(1)–Cl(1)<br>Mn(1)–Cl(1)a<br>Mn(1)–Cl(1)c<br>Mn(1)–Cl(1)d	2.268(5)<br>2.268(5)<br>2.5501(14)<br>2.5501(14)<br>2.6129(14)<br>2.6129(14)	N(1)–Mn(1)–N(1)a<br>N(1)–Mn(1)–Cl(1)<br>N(1)c–Mn(1)–Cl(1)c<br>N(1)a–Mn(1)–Cl(1)a<br>N(1)d–Mn(1)–Cl(1)d<br>N(1)a–Mn(1)–Cl(1)<br>N(1)a–Mn(1)–Cl(1)c<br>N(1)a–Mn(1)–Cl(1)a<br>Cl(1)–Mn(1)–Cl(1)c<br>Cl(1)–Mn(1)–Cl(1)a<br>Cl(1)–Mn(1)–Cl(1)d<br>Cl(1)c–Mn(1)–Cl(1)a<br>Cl(1)c–Mn(1)–Cl(1)d<br>Cl(1)a–Mn(1)–Cl(1)d<br>Cl(1)d–Mn(1)–N(1)a	180.00<br>90.43(13)<br>92.96(13)<br>89.57(13)<br>87.05(13)<br>89.57(13)<br>87.05(13)<br>90.43(13)<br>93.23(4)<br>180.00<br>86.77(4)<br>86.77(4)<br>180.00<br>93.23(4)<br>92.96(13)

* Symmetry code: (a): 1-*x*,1-*y*,-*z*; (b): -*x*,1-*y*,1-*z*; (c): -1+*x*, *y*, *z*; (d): 2-*x*,1-*y*,-*z*; (e): 1-*x*,*y*,3/2-*z*

Table S3. Doubled chloro-bridged manganese(II) coordination polymers (Cambridge Structure Database, 1-17)

**Compound**	**Mn-N [Å]**	**Mn-Cl [Å]**	**Mn···Mn [Å]**	**Ref.**
$[Mn(btzh)Cl_2]_n$	2.305(3)<br>2.305(3)	2.559(1)<br>2.560(1)	3.728	1
$[Mn(btzh)Cl_2]_n$	2.282(3)<br>2.280(3)	2.5591(1)<br>2.5522(11)	3.6906	2
$[Mn(im)Cl_2]_n$	2.201(1)<br>2.201(1)	2.595(2)<br>2.595(2)	3.761	3
$[Mn(mapy)Cl_2]_n$	2.260(1)<br>2.303(2)<br>2.284(1)<br>2.279(1)	2.4978(8)<br>2.6102(8)<br>2.5583(8)<br>2.6259(7)	3.7964<br>3.7614	4
$[Mn(phen)Cl_2]_n$	2.256(2)<br>2.256(2)	2.650(1)<br>2.650(1)	3.8172	5
$[Mn(phen)Cl_2]_n$	2.2608(10)	2.4809(3)	3.8203	6

	2.2608(10)	2.6630(3)		
$[Mn(NCMe)_2Cl_2]_n$	2.243	2.5396(7)	3.743	7
$[Mn(4\text{-}CNpy)Cl_2]]_n$	2.285(7)<br>2.285(7)	2.525(2)<br>2.525(2)	3.700	8
$[Mn(amtet)Cl_2]]_n$	2.266<br>2.266	2.5417<br>2.5395	3.6256	9
$[Mn(tmen)Cl_2)_n$	2.362(2)<br>2.362(2)<br>2.360(2)<br>2.360(2)	3.941(1)<br>2.608(1)<br>2.360(2)<br>2.600(1)	3.941<br>3.940	10
$[Mn(H_2dapd)_2Cl_2]_n$	2.39(1)<br>2.34(1)	2.468(1)<br>2.759(1)<br>2.580(1)<br>2.829(1)	4.050	11
$[Mn(H_2dapd)_2Cl_2]_n$	2.324(1)<br>2.369(1)<br>2.369(1)	2.526(2)<br>2.794(2)	4.050	12
$[Mn(pyam)_2Cl_2]_n$	2.281(2)<br>2.280(2)	2.4749(5)<br>2.5724(7)<br>2.6914(7)<br>2.4617(6)	3.7479	13
$[Mn(ammepyr)_2Cl_2]_n$	2.316<br>2.316	2.5415<br>2.5950	3.7425	14
$[Mn(bethz)_2Cl_2]_n$	2.307 (4)<br>2.307 (4)	2.307 (4)<br>2.5232 (10)	3.585	15
$[Mn(bpy)Cl_2]_n$	2.258(3)	2.481(1)<br>2.662(1)	3.835	16
$[Mn(py)Cl_2]_n$	2.201	2.592(2)<br>2.594(2)	3.761	3

btzh = benzotriazole; *im*= imidazole; *mapy* = 2-(methylamino)pyridine, *phen* = 1,10-phenantroline; *NCMe* = acetonitrile; *4-CNpy* = 4-cyanpyridine, *amtet* = 5-amino-1-H-tetrazole; *tmen* = N,N,N',N'-tetramethylethylenediamine, *H_2dapd* = 2,6-diacetylpyridine dioxime; *pyam* = benzyl(2-pyridylmethylene)amine); *ammepyr* = 4-amino-4-methylpyrimidine); *beth* = 1,3-benzothiazole, *bpy* = 2,2'-bipyridine; *py* =pyridine

references (Ref) in Table S3:

1. I. Sotofte, K. Nielsen, Acta Chemica Standinavica, 1984, 257, 257
2. F. A. Brede, F. Mühlbach, G. Sextl, K.Müller-Buschbaum, Dalton Trans., 2016, 45, 10609
3. S.Gorter, A.D. van Ingen Schenau, G.C.Verschoor, Acta Crystallogr.,Sect. B: Struct. Crystallogr. Cryst. Chem., 1974, 30, 1867
4. D. Domide, O. Hübner, S.Behrens, O.Walter, H. Wadepohl, E.Kaifer, H.-J. Himmel, Eur. J. Inorg. Chem. 2011, 1387–1394
5. A. Majumder, M. Westerhausen, A.N. Kneifel, J.-P. Sutter, N. Daro, S. Mitra, Inorg.Chim.Acta, 2006, 359, 3841
6. X.-M. Lu, P.-Z. Li, X.-T. Wang, S. Gao, X.-J. Wang, L. Zhou, C.-S.Liu, X.-N. Sui, J.-H. Feng, Y.-H. Deng, Q.-H. Jin, J. Liu, N. Liu, J.-P. Lian, Polyhedron, 2008, 27, 3669
7. K. I. Pokhodnya, M. Bonner, A.G. DiPasquale, A. L. Rheingold, J.-H. Her, P. W. Stephens, J.-W. Park, B. S. Kennon, A.M. Arif, J. S. Miller, Inorg. Chem.,2007, 46, 2471
8. W. Zhang, J.R. Jeitler, M.M. Turnbull, C.P. Landee, M. Wei, R.D. Willett, Inorg. Chem. Acta, 1997, 183, 256

9. E.A. Buvaylo, V.N. Kokozay, O.Yu. Vassilyeva, B.W. Skelton, M.M. Degtyarik, M. Korabik, J. Jezierska, J.Coord.Chem., 2015, 68, 1261
10. P.Sobota, J.Utko, S.Szafert, Z.Janas, T.Glowiak, J.Chem.Soc.,Dalton Trans., 1996, 3469
11. B.C.Unni Nair, J.E.Sheats, R.Ponteciello, D.Van Engen, V.Petrouleas, G. C. Dismukes, Inorg Chem., 1989, 28, 1582
12. R.E.Marsh., Inorg Chem., 1980, 29, 572
13. Y. Song, Z. Xu, Q. Sun, B. Su, Q. Gao, H. Liu, J.Zhao, J. Chem. Coord., 2007, 60, 2351
14. K.A. Vinogradova, N.A. Shekhovtsov, A.S. Berezin, T.S. Sukhikh, V.P. Krivopalov, E.B. Nikolaenkova, I.V. Plokhikh, M.B. Bushuev, Inorg. Chem. Commun., 2019, 100, 11
15. H. Bouchareb, S. Benmebarek, S. Bouacida, H. Merazig, M. Boudraa, Acta Crystallogr., Sect.E: Struct. Rep. Online, 2014, 70, m275
16. M. Lubben, A. Meetsma, B.L. Feringa, Inorg. Chem. Acta, 1995,230,169

Table S4. Short intra– and intermolecular contacts in **1**–**3**.

D—H•••A	D—H	H•••A	D•••A [Å]	D—H•••A [°]
		**1**		
O(1)–H(1)•••Cl(1)	0.82	2.39	3.1520(15)	155.00
		**2**		
O(1)–H(1)•••Cl(1)	0.85	2.36(3)	3.159(4)	157(4)
		**3**		
C(2)–H(2)•••Cl(1)	0.93	2.68	3.388(7)	134.00
C(5)–H(5)•••Cl(1)	0.93	2.81	3.627(7)	147.00

Table S5. Short π•••π stacking interactions in **1–3**.

Cg(I)•••Cg(J)	Cg(I)•••Cg(J) [Å]	α [°]	β [°]	γ [°]	Cg(I)-Perp [Å]	Cg(J)-Perp [Å]
			**1**			
Cg(1)•••Cg(2)[a]	3.6782(12)	0.63(11)	26.74	26.22	-3.2996(8)	-3.2849(8)
Cg(1)•••Cg(2)[b]	3.5524(12)	0.63(11)	21.14	21.73	3.2999(8)	3.3134(8)
Cg(2)•••Cg(2)[a]	3.4020(12)	0	14.48	14.48	-3.2939(8)	-3.2939(8)
			**2**			
Cg(3)•••Cg(4)[c]	3.598(3) Å	0.7(3)	21.92	22.57	-3.322(2)	-3.337(2)
Cg(3)•••Cg(4)[d]	3.673(3) Å	0.7(3)	26.58	25.98	3.301(2)	3.284(2)
Cg(4)•••Cg(4)[d]	3.425(3)	0	15.93	15.93	3.293(2)	3.294(2)
			**3**			
Cg(5)•••Cg(6)[e]	3.731(4)	0.8(4)	17.88	17.56	-3.558(3)	3.552(3)

*α = dihedral angle between *Cg*(I) and *Cg*(J); *Cg*(I)-Perp = Perpendicular distance of *Cg*(I) on ring J; *Cg*(J)-Perp = perpendicular distance of *Cg*(J) on ring I; β = angle *Cg*(I)→*Cg*(J) vector and normal to ring I; γ = angle *Cg*(I) →*Cg*(J) vector and normal to plane J;

Cg(1): N(1)/C(1)/N(2)/C(6)/C(7)
Cg(2): N(2)/C(1)/C(2)/C(3)/C(4)/C(5)
Cg(3): N(1)/C(1)/C(2)/N(2)/C(7)
Cg(4): N(2)/C(3)/C(4)/C(5)/C(6)/C(7)
Cg(5): N(1)/C(1)/N(2)/C(6)/C(7)
Cg(6): N(2)/C(1)/C(2)/C(3)/C(4)/C(5)

*Symmetry code: (a) -*x*,1-*y*,1-*z*; (b): 1-*x*,1-*y*,1-*z*; (c) -*x*,1-*y*,-*z;* d): 1-*x*,1-*y*,-*z*; (e): 1+*x*, *y*, *z*

Table S6.Thermoanalytical data for complexes **1-3**.

		Thermogravimetry		
	DTG Peak	***Weight loss [%]***		***Decomposition product***
Complex	T [^{o}C]	***Found***	***Calcd***	
1	234	*26*	*21.6*	*-Cl_2*<br>*-$2H_2O$*
	334	*21.8*	*25.6*	*-Cl_2*<br>*-$2N_2$*
	381<br>603	*18.2*<br>*14.2*	*35.2*	*-2 impy*
**Total for** 1**:**		***80.2***	***82.4***	***MnO_2 as residue product***
2	83	*5.2*	*6.2*	*-$2H_2O$*
	229	*29.6*	*27.4*	*-Br_2*
	383	*10.6*	*12.1*	*-Cl_2*
	362<br>576	*28.9*<br>*11.8*	*39.4*	*-2 impy*
**Total for** 2**:**	**-**	***86.1***	***85.1***	***MnO_2 as residue product***
3	210<br>290	*21.0*<br>*17.4*	*19.6*<br>*15.5*	*- Cl_2*<br>*-$2N_2$*
	416<br>650	*20.7*<br>*14.9*	*40.9*	*- impy*
**Total for** 3**:**	**-**	***73.9***	***76.0***	***MnO_2 as residue product***

Table S7. The AutoDock4 scores of binding [kcal/mole] of the studied manganese complexes (**1-3**) to selected binding sites of the human serum albumin. Site numbering as in Figure 7 (see paper). The scores within kT from the best result shown on gray background.

**1**		**2**		**3**	
**Site #**	**Score**	**Site #**	**Score**	**Site #**	**Score**
2	−6.33	2	−6.58	1	−4.92
1	−6.24	1	−6.35	1	−4.66
1	−6.15	1	−6.29	2	−4.44
1	−6.02	2	−5.73	2	−4.44
2	−5.64	2	−5.47	2	−4.13
2	−5.29	2	−5.11	2	−4.12
2	−4.98	2	−5.07	2	−3.99
2	−4.92			2	−3.96
				2	−3.86

Table S8. The AutoDock4 scores of binding [kcal/mole] of the studied manganese complexes **1-3** to selected binding sites of the apo structure of the human serum apotransferrin apo-Tf. Site numbering as in Figure 1(see paper). The scores within kT from the best result shown on gray background.

3		1		2	
**Site #**	**Score**	**Site #**	**Score**	**Site #**	**Score**
5	-4.19	5	-5.45	5	-5.48
5	-4.11	5	-5.2	5	-5.35
1	-3.93	5	-5.11	5	-5.16
5	-3.84	4	-4.69	2	-4.8
4	-3.71	2	-4.68	2	-4.74
2	-3.69	2	-4.66	4	-4.74
2	-3.63	4	-4.6	4	-4.73
4	-3.61	4	-4.6	4	-4.71
4	-3.6	4	-4.58	2	-4.47
2	-3.59	4	-4.47	4	-4.39
4	-3.56	2	-4.38	2	-4.37
4	-3.49	2	-4.34	2	-4.35
2	-3.4	2	-4.27	2	-4.34
2	-3.09	2	-4.23	2	-4.32
2	-2.92	1	-4.17	2	-4.17
2	-2.9	2	-4.13	2	-4.11
2	-2.9	2	-4.12	1	-4.05
2	-2.9	4	-4.1	1	-3.96
3	-2.67	1	-3.99	1	-3.96
		1	-3.97	2	-3.94
		2	-3.86	2	-3.91
		3	-3.69	2	-3.9
		3	-3.58	3	-3.79
		3	-3.41	3	-3.45
